# Electrochemical intercalation and exfoliation of (Sb_2_Te_3_)_2_(Sb_2_)_2_

**DOI:** 10.1039/d6ra02757a

**Published:** 2026-09-01

**Authors:** Tung T. Nguyen, Yimo Hou, Selene Koremenos-Tsebelis, Nishkarsh Agarwal, Robert Hovden, Pierre Ferdinand P. Poudeu, Stephen Maldonado

**Affiliations:** a Department of Chemistry, University of Michigan Ann Arbor Michigan 48109-1055 USA smald@umich.edu; b Program in Applied Physics, University of Michigan Ann Arbor Michigan 48109-1055 USA; c Material Science and Engineering, University of Michigan Ann Arbor MI 48109 USA

## Abstract

An electrochemical method for exfoliating (Sb_2_Te_3_)_2_(Sb_2_)_2_, a van der Waals material, topological insulator, and the first member of the homologous series of topological superlattices (Sb_2_Te_3_)_*m*_(Sb_2_)_*n*_ with *m* = *n* has been demonstrated. The objective of this work was to determine whether electrochemical exfoliation can yield pristine few or single layer 2D materials in this material system. To this end, polycrystalline ingots of (Sb_2_Te_3_)_2_(Sb_2_)_2_ were prepared from stoichiometric mixtures of elemental Sb and Te heated in vacuum. Diffraction and scanning transmission electron microscopy data confirmed the 2/2 stacking sequence. Cathodic exfoliation was performed and studied using 4 separate cations, H^+^, Na^+^, Li^+^, and tetrapropylammonium (TPA^+^). Delamination of the surface of (Sb_2_Te_3_)_2_(Sb_2_)_2_ electrodes was consistently observed at negative applied biases. These materials were collected and characterized *via* scanning electron microscopy, transmission electron microscopy, atomic force microscopy, and Raman spectroscopy. The delaminated materials were layered, consistent with exfoliation of the parent ingot. The key findings were that materials prepared by electrochemical exfoliation were less prone to oxidation in air than layer stacks obtained by mechanical exfoliation and that cathodic exfoliation performed with larger cations yielded a more homogeneous, single-crystalline structure as compared to the smaller cations. These differences indicated the selectivity of cleavage planes for electrochemical exfoliation *vs.* mechanical exfoliation. The significance of these findings will be to prepare thin materials that help understand how the electronic properties of topological insulators evolve with the number of layers.

## Abstract 2

An electrochemical method for exfoliating (Sb_2_Te_3_)_2_(Sb_2_)_2_, a van der Waals material, topological insulator, and the first member of the homologous series of topological superlattices (Sb_2_Te_3_)_*m*_(Sb_2_)_*n*_ with *m* = *n* has been demonstrated.

## Graphical abstract

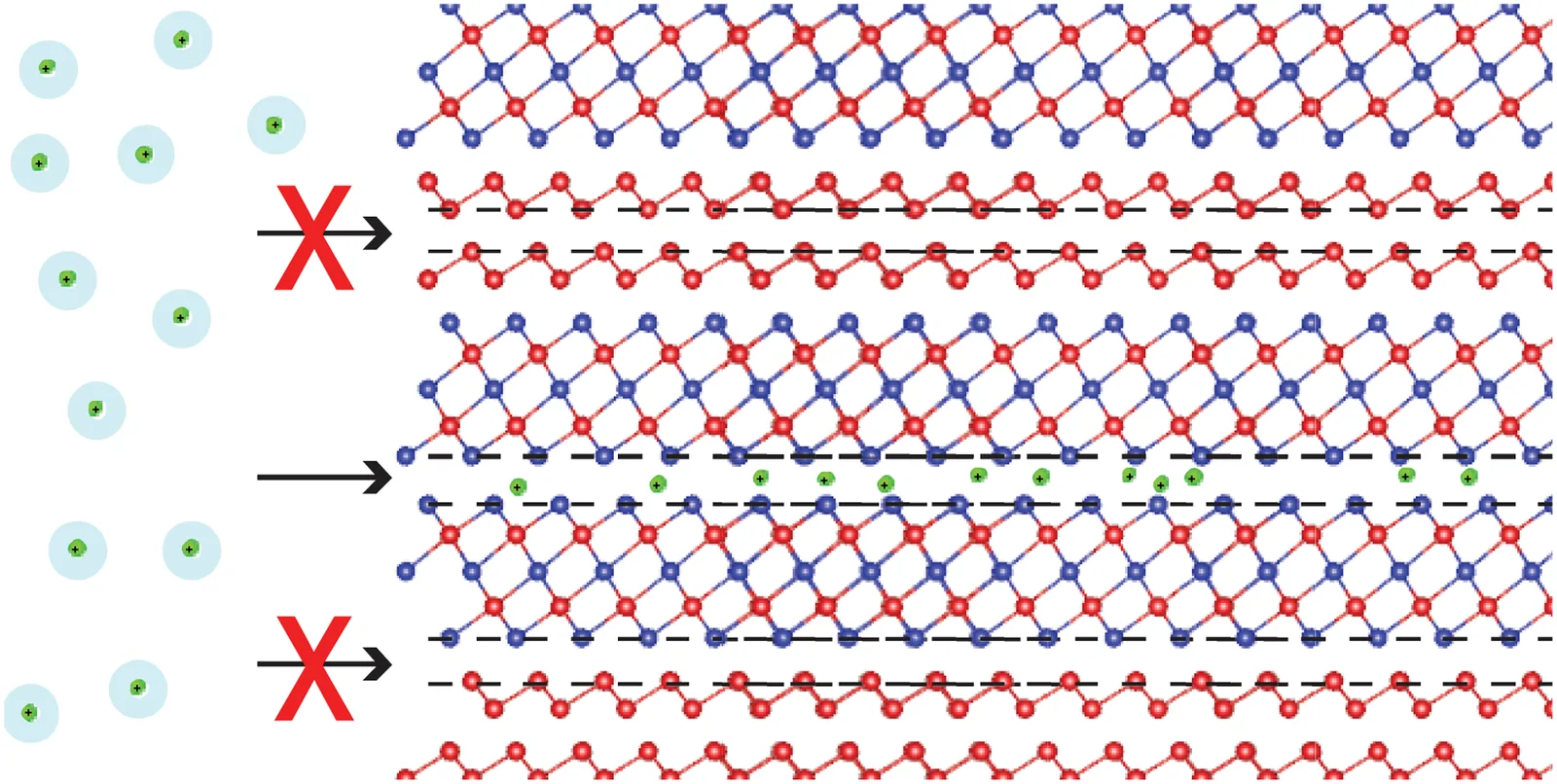

## Introduction

Two-dimensional (2D) transition metal dichalcogenide (TMD) materials, a subclass of van der Waals heterostructures, include covalently bonded MX_2_ layers (where M is the transition metal and X is a chalcogen atom) that are bound by weak van der Waals force. Among them, antimony telluride (Sb_2_Te_3_) is a low-temperature thermoelectric material with a rhombohedral crystalline structure.^1^ Due to having a high thermoelectric figure of merit, antimony telluride is considered potentially useful for direct energy conversion. A particularly attractive feature of Sb_2_Te_3_ is that a related set of natural superlattices^2^ can be realized by insertion of additional semi-metallic Sb_2_ layers to form ordered stacking sequences with different charge transport and thermoelectric properties. This homologous series of compounds is denoted as (Sb_2_Te_3_)_*m*_(Sb_2_)_*n*_.^3^

(Sb_2_Te_3_)_1_(Sb_2_)_1_ is hypothetically the simplest member of these homologous misfit materials. In principle, synthesis of this material with a 4 : 3 atomic ratio of Sb and Te source elements yields an atomic structure with the simplest repeating unit of 1 quintuple layer of Sb_2_Te_3_ adjacent to 1 bilayer of Sb_2_, *i.e.* (Sb_2_Te_3_)_1_(Sb_2_)_1_.^1,4^ However, all sets of (Sb_2_Te_3_)_*m*_(Sb_2_)_*n*_ materials where *m* = *n* do not have the same atomic structures even though they can be described coarsely as ‘Sb_4_Te_3_’. For example, (Sb_2_Te_3_)_2_(Sb_2_)_2_ has a lattice structure with two quintuple layers of Sb_2_Te_3_ adjacent to two Sb_2_ layers.^5,6^ In terms of electronic properties, the atomic structure of (Sb_2_Te_3_)_2_(Sb_2_)_2_ results in topological insulating layers of Sb_2_Te_3_ and Sb_2_, in which there is the presence of a narrow band gap, protected by conducting surface edge states.^2,7^ It is presently unclear what, if any, consequences this atomic structure has on the evolution of electronic properties of materials in few or single layers of (Sb_2_Te_3_)_2_(Sb_2_)_2_.^2^

The electrochemical behavior of (Sb_2_Te_3_)_*m*_(Sb_2_)_*n*_ materials is thus of interest because electrochemical exfoliation is one desirable method to cleave layered materials into thin, 2D flakes. In general, solution-phase exfoliation methods are attractive over the traditional ‘tape’ method due to the possibility of yielding large quantities of 2D materials.^8^ Purely-chemical routes like the direct liquid-phase exfoliation method lack control over the thickness and uniformity of the 2D materials.^9^ In contrast, electrochemical exfoliation by intercalation have the potential to yield single-or-few layer thick materials by insertion of guest ionic species into the van der Waals gaps,^9,10^ with the magnitude and polarity of the applied potential dictating whether anions or cations intercalate into the bulk materials. Although anodic exfoliation (positive bias) often has a high exfoliation efficiency, extreme positive potentials are intrinsically destructive and often result in highly oxidized materials.^11^ Even if the exfoliated material is natively stable at positive applied potentials, reactive oxygen species formed simultaneously can chemically attack the exfoliated materials.^12^ Hence, cathodic intercalation at negative applied bias, which avoids reactive oxygen species, is potentially more desirable for yielding non-oxidized and functional 2D nanosheets from the bulk structure.

Although the electrochemical exfoliation of Sb_2_Te_3_ and Sb_2_ is known,^13,14^ the electrochemical exfoliation of (Sb_2_Te_3_)_2_(Sb_2_)_2_ is not known. Herein, we report the cathodic intercalation of cations into (Sb_2_Te_3_)_2_(Sb_2_)_2_ ingot electrodes using a three electrode cell operating under potentiostatic control. Specifically, understanding and identifying the electrochemical behavior of (Sb_2_Te_3_)_2_(Sb_2_)_2_ is necessary to eventually exfoliate mono-to-few layers of (Sb_2_Te_3_)_*m*_(Sb_2_)_*n*_. The data below are presented and discussed in this context.

## Experimental

### Materials and chemicals

Antimony (200 mesh, 99.5%, Alfa Aesar), tellurium (100 mesh, 99.999%, Thermo Scientific), copper wire (0.5 mm, Hobbyworker), conductive silver paint (SPI supply), Tetra-*n*-propylammonium chloride (99%, Alfa Aesar), hydrochloric acid (Certified ACS Plus, 36.5 to 38.0%, ThermoFisher), sodium chloride (≥99.0%, Sigma Aldrich) methanol (ACS grade, BDH), epoxy adhesive (Loctite), were used as received. Nanopure water (>18.2 MΩ cm, Barnstead Nanopure) was used throughout.

### Bulk ingot synthesis

Sb and Te powders were weighed according to the desired stoichiometric ratios in an Ar-filled glovebox (O_2_/H_2_O < 1 ppm), and pre-ground for ∼10 min to reduce the particle size and improve mixing. The blended powders were loaded into quartz tubes (7 mm ID, 9 mm OD), evacuated to ∼1 × 10^−3^ Torr, and flame-sealed. The tube furnace was preheated to 720 °C prior to loading, and the sealed tubes were vertically inserted and melted at 720 °C for 20–30 min. This melting step was repeated five times while flipping the direction of the tube, to ensure complete homogenization of the ingots. The samples were then annealed at 550 °C for two months to promote atomic ordering and phase stability, followed by slow cooling to room temperature over ∼3 h.

### Polycrystalline ingot electrode assembly

Silver paint and a droplet of eutectic gallium indium were used to connect the as-prepared ingot of either Sb_2_Te_3_, (Sb_2_Te_3_)_2_(Sb_2_)_2_, or Sb_2_ with copper electrical wire. The ohmicity of this type of electrical contact to the ingot electrode was verified by collecting current–voltage profiles on test pieces with two symmetric contacts that obeyed Ohm's law. For electrode formation, the copper wire was threaded through a glass rod and the ingot was affixed to the end with Loctite (Hysol 1C) epoxy. This epoxy was necessary to prevent liquid infiltration that would compromise the ohmic contact and/or expose the copper wire.

### Mechanical exfoliation

Bulk ingots of Sb_2_Te_3_, Sb_2_, and (Sb_2_Te_3_)_2_(Sb_2_)_2_ were mechanically exfoliated into thin flakes using the ‘scotch tape ‘method.^15^ Specifically, Scotch tape (Magic tape, 3 M) was pressed onto a crystal surface and peeled vertically to delaminate the flakes. The tape was then folded so that both edges of the crystal were adhered to the tape. Then the tape unfolded to mechanical cleave between the van Waals gap and expose thin layers of materials. Folding and unfolding was repeated many times to yield thinner mechanically exfoliated flakes. These flakes were then transferred to a support substrate for subsequent measurement.

### Electrochemistry

Electrochemical intercalation and exfoliation experiments were performed in a glass beaker with a Solartron 1176 A potentiostat, an ingot electrode, a Pt counter electrode, and a Ag/AgCl reference electrode in roughly 10–15 mL of aqueous solution (Fig. 2a). The separation distance between working and counter electrodes was 1.5 cm. All reported potentials are with respect to *E*(Ag/AgCl, satd. KCl). Intercalation and exfoliation were performed at *E* = −2.0 V for 10 minutes. All the electrochemistry data were normalized by a rough geometric area of the surface of ingot working electrode. During the applied bias, materials were delaminated readily from the host ingot electrode. These materials were collected, washed, and centrifuged in nanopure water for 3 times before being bath-sonicated in isopropyl alcohol for 20 minutes to further break down the intercalated flakes from the solution. The final product of the exfoliation was an “ink” solution, which contained exfoliated (Sb_2_Te_3_)_2_(Sb_2_)_2_. All electrochemical intercalation experiments/measurements were performed in triplicate, with a different ingot electrode for each replicate.

### Material characterization

Ink solutions of exfoliated (Sb_2_Te_3_)_2_(Sb_2_)_2_ were drop cast onto different support substrates for further analysis, including Si(111) wafer (University Wafer) sections and 200-mesh carbon film on copper support transmission electron microscopy (TEM) grid (Ted Pella).

Scanning electron microscopy (SEM) and accompanying energy-dispersive spectroscopy (EDS) elemental mapping were conducted using a JEOL JSM-7800FLV field emission SEM equipped with an Everhart–Thornley secondary electron detector, operated at an accelerating voltage of 18 kV.

Bright-field transmission electron microscopy (TEM) and selected area electron diffraction (SAED) measurements of exfoliated flakes were performed using a Thermo Fisher Talos F200X G2 S/TEM. Data were collected with Thermo Scientific Velox Software and a Gatan One View bottom mount camera.

Samples for atomic-resolution high-angle annular dark-field scanning transmission electron microscopy (HAADF-STEM) were prepared using a Thermo Fisher Scientific (TFS) Helios 650 nanolab focused ion beam (FIB) using the standard lift-out method. A 200 nm thick carbon layer and a 1 µm thick Pt layer were deposited as a protective capping layer on the flakes mechanically exfoliated on a Si substrate before lift-out. A final polish with a 2 kV Ga ion was performed to mitigate any surface damage. Imaging of cross-sectional samples of (Sb_2_Te_3_)_2_(Sb_2_)_2_ ingot sections was performed with an aberration-corrected TFS Spectra 300 operated at 300 kV with a 30 mrad convergence angle. The HAADF inner and outer collection angles were 60 and 200 mrad, respectively, and acquired as a series of 50 rapid-frame images (500 ns each) which were then subsequently realigned and averaged using the rigid registration method.^16^ A high-fidelity and high signal-to-noise image of the atomic lattice was obtained. A minimal bandpass filter was used for background subtraction. Atomic column positions were first identified with sub-pixel peak fitting using the open source ‘Atomap’ package.^17^ The extracted atomic columns were projected along the direction perpendicular to the layers. The projected coordinates were sorted. Individual layers were identified by detecting gaps between clusters of closely spaced values, with the mean position of each layer obtained by averaging coordinates within each cluster. Interlayer spacings were calculated from the differences between consecutive layer positions.

X-Ray diffraction patterns were collected using a Rigaku SmartLab diffractometer equipped with a Cu Kα X-ray source, operated at 40 kV and 44 mA. Instrumental broadening parameters for this SmartLab system were obtained using the Whole Powder Pattern Fitting (WPPF) module in the SmartLab Studio II Guidance software, which was also used to analyze and refine the data.

Atomic force microscopy (AFM) measurements were characterized using a Veeco Dimension Icon AFM. The instrument was equipped with silicon nitride Scanasyst-Air tips (tip radius: 2 nm, length: 115 µm, width: 25 µm, resonant frequency: 70 kHz). Images were acquired using PeakForce Tapping mode and processed using Bruker Nanoscope Analysis software.

Raman spectra were obtained with a confocal Renishaw InVia Raman microscope equipped with a Nikon LU Plan 20× objective (NA = 0.4) and a point source 785 nm diode laser. For reference samples, mechanical exfoliation was performed with adhesive tape immediately prior to interrogation.

## Results

### Structural characterization of bulk ingot

**Fig. 1:**
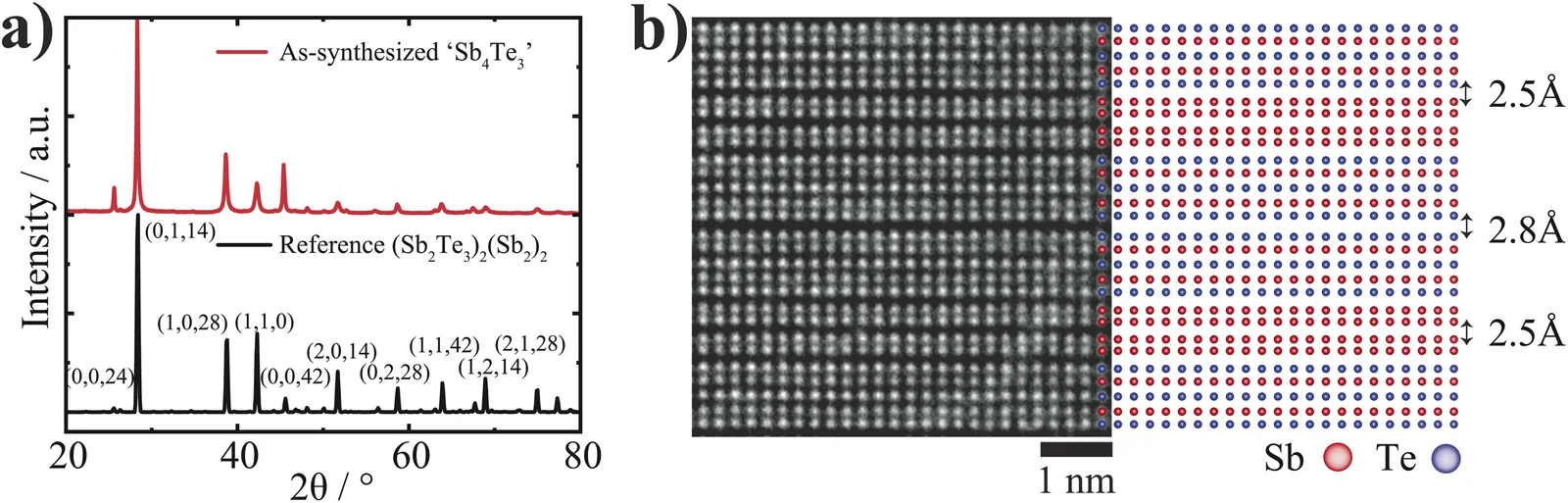
(a) X-ray diffraction of as-synthesized (Sb_2_Te_3_)_*m*_(Sb_2_)_*n*_ with *m* = *n* ingot with the corresponding reference powder X-ray diffraction data from Inorganic Crystal Structure Database. (b) Representative high – angle annular dark-field scanning transmission electron microscopy (HAADF-STEM) image of a cross-sectioned sample of the ingot viewed along the 11̄0 direction. The cross-sectional image illustrates the alternate stacking of 2 quintuple Sb_2_Te_3_ and 2 bilayers Sb_2_. Sb and Te atoms were denoted with red and purple circles, respectively. The measured gap spacings between van der Waals layers of Sb_2_Te_3_ and Sb_2_ sub-units are indicated.

As-synthesized (Sb_2_Te_3_)_*m*_(Sb_2_)_*n*_ (*m* = *n*) ingots were examined by X-ray diffraction (Fig. 1a). The diffraction pattern contained several peaks that could all be indexed to reference data for polycrystalline (Sb_2_Te_3_)_*m*_(Sb_2_)_*n*_ (*m* = *n*) and were distinct from the X-ray diffraction data of as-synthesized Sb_2_Te_3_ and Sb_2_ (SI, Fig. S1). The diffraction peaks in Fig. 1a were consistent with a *c* parameter of 83.46 Å, equivalent to the sum of 2 quintuple Sb_2_Te_3_ (*c* = 30.46 Å) and 2 bilayers Sb_2_ (*c* = 11.27 Å), *i.e. m* = *n* = 2, and in agreement with the reported value of 83.56 Å for (Sb_2_Te_3_)_2_(Sb_2_)_2_.^5^ To separately assess the atomic structure of the as-prepared (Sb_2_Te_3_)_*m*_(Sb_2_)_*n*_ (*m* = *n*), thin samples were obtained by focused ion beam milling and analyzed by high-angle annular dark-field scanning transmission electron microscopy (HAADF-STEM). A section of the ingot surface was cut and transferred to a polished Si substrate. Focused ion beam milling was conducted parallel to the edge of the section. Milling was performed vertically for cross sections. Fig. 1b shows representative data, viewed along the 11̄0 direction. The contrast between Sb and Te in HAADF-STEM was negligible because of the similarity of atomic numbers. Nevertheless, the quintuple layers of Sb_2_Te_3_ and Sb_2_ bilayers were identifiable along the 11̄0 direction with minimal lattice mismatching. In this direction, AA′B′ABB′C′BCC′A′C stacking of one unit cell could not be directly observed. However, the viewing perspective was parallel to the plane of the layers, allowing direct visualization of the gaps between layers. The HAADF-STEM data directly confirmed the stacking sequence as (Sb_2_Te_3_)_2_(Sb_2_)_2_ with exactly three different layer gap types: Sb_2_Te_3_/Sb_2_Te_3_, Sb_2_/Sb_2_, and Sb_2_Te_3_/Sb_2_. The measured gaps were 2.80 ± 0.02 Å, 2.51 ± 0.03 Å, and 2.51 ± 0.03 Å, respectively. Notably, the Sb_2_Te_3_/Sb_2_ gaps measured here fell within the prior reported values of 2.3 Å and 2.6 Å, respectively.^5,6^ Each of these gaps represents a potential insertion site for ion intercalation.

### Electrochemistry

**Fig. 2:**
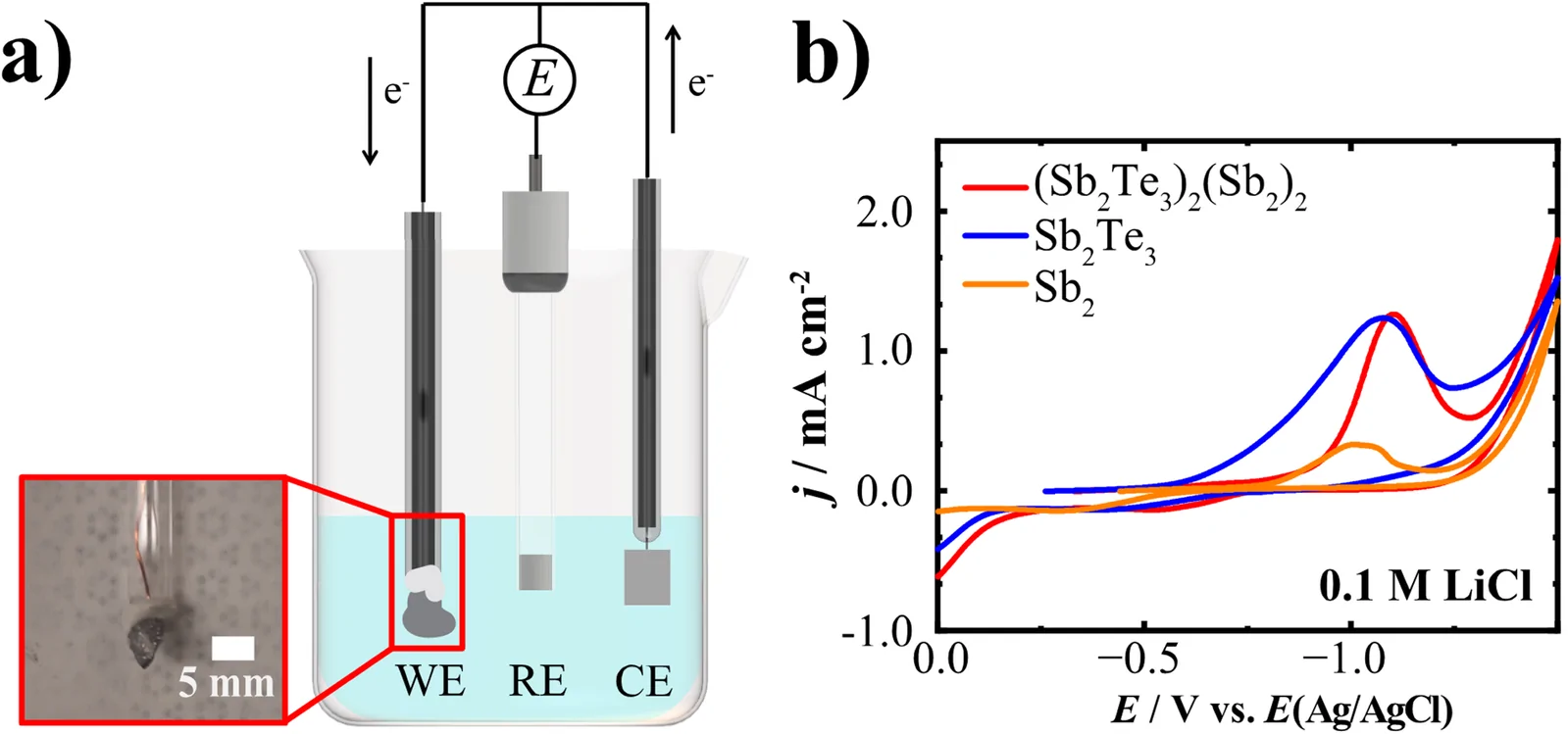
(a) Schematic depiction of electrochemical intercalation and exfoliation of a (Sb_2_Te_3_)_2_(Sb_2_)_2_ ingot working electrode in a 3-electrode cell. Inset: a representative photograph of an ingot electrode used in this work. (b) Voltammetric responses for (Sb_2_Te_3_)_2_(Sb_2_)_2_, Sb_2_Te_3_, and Sb_2_ electrodes at 10 mV s^−1^ in 0.1 M LiCl(aq).

Cyclic voltammetry was performed in 0.1 M LiCl(aq) with (Sb_2_Te_3_)_2_(Sb_2_)_2_, Sb_2_Te_3_, and Sb_2_ ingot electrodes (Fig. 2b). Each electrode type exhibited a pre-wave at potentials slightly more positive than the onset of H_2_(g) evolution at −1.4 V. The pre-wave centered at −1.0 to −1.1 V was consistent with prior reports of Sb^3+^ reduction to Sb^0^.^18,19^ The total cathodic charge passed in this voltammetric feature was noticeably larger for (Sb_2_Te_3_)_2_(Sb_2_)_2_ and Sb_2_Te_3_ (0.31 and 0.44 mC cm^−2^, respectively, *vs.* 0.085 mC cm^−2^ for Sb_2_). The integrated charge for Sb_2_ was in line for an adsorbed redox process (*e.g.* reduction of surface oxide), *i.e.* 0.01–0.1 mC cm^−2^,^20^ but the larger integrated charge densities for (Sb_2_Te_3_)_2_(Sb_2_)_2_ and Sb_2_Te_3_ implied redox activity that extended below the surface. This charge was also nominally insensitive to the anion identity, as essentially identical voltammograms were recorded with sulfate and perchlorate electrolytes. The Sb^3+^ reduction peak currents were similar in magnitude for (Sb_2_Te_3_)_2_(Sb_2_)_2_ and Sb_2_Te_3_ (1.26 mA cm^−2^*vs.* 1.23 mA cm^−2^, respectively) while the peak currents were notably smaller with Sb_2_ electrodes (0.33 mA cm^−2^). This latter observation was consistent with Sb^3+^ being present in the form of surface oxide(s) in Sb_2_. The onset of cathodic current was noticeably more positive for Sb_2_Te_3_ electrodes. For all three electrode materials, very little oxidative current passed in the potential range shown in Fig. 2b, indicating an irreversible process. Visually, a thin stream of ejected material was observed for all three materials during the cathodic sweep for potentials at or more negative than −1.0 V, albeit the amount of material for Sb_2_ was slight in comparison to the materials streams for (Sb_2_Te_3_)_2_(Sb_2_)_2_ and Sb_2_Te_3_.

**Fig. 3:**
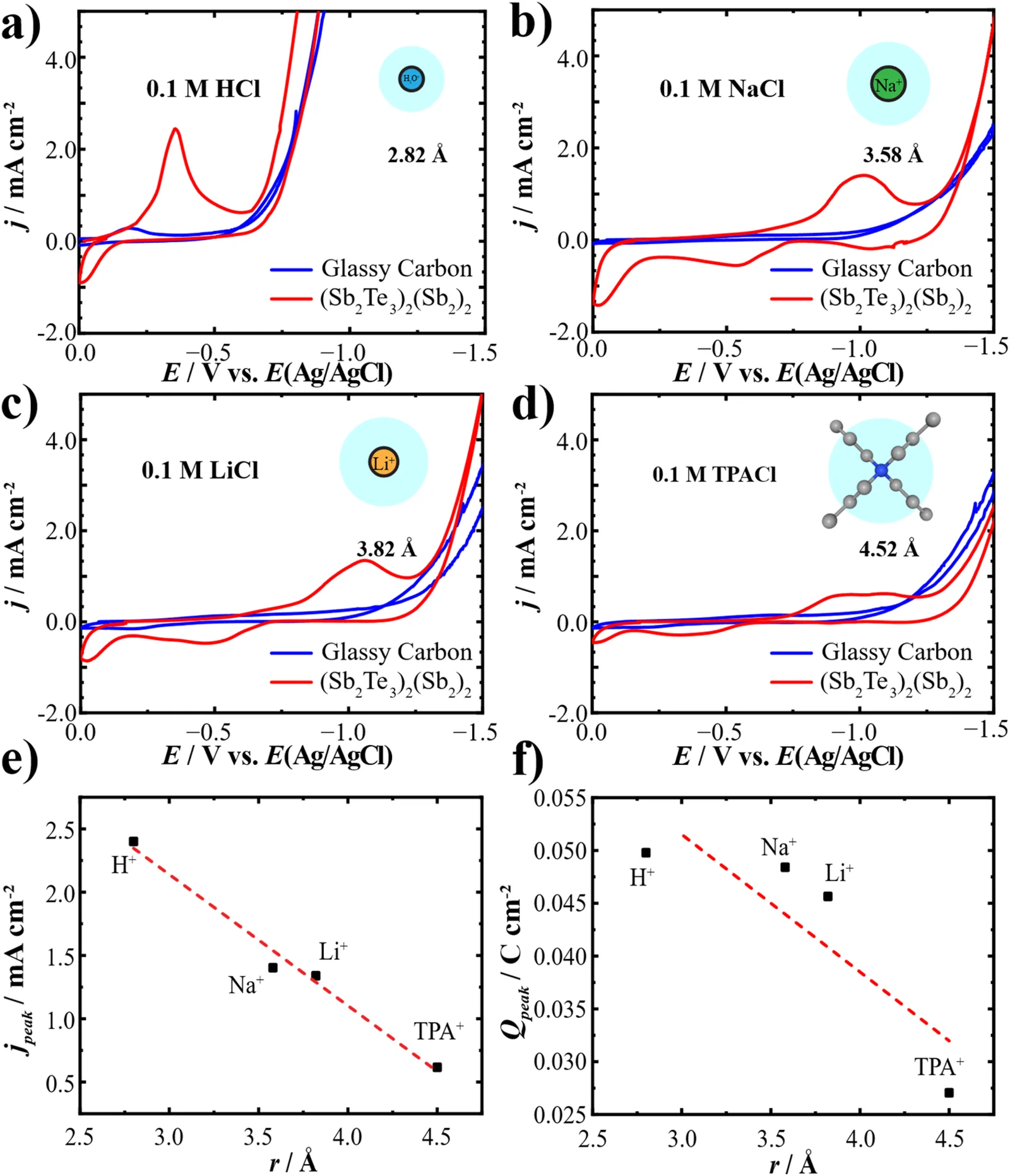
Voltammetric responses for (Sb_2_Te_3_)_2_(Sb_2_)_2_ electrodes and glassy carbon electrodes at 10 mV s^−1^ in (a) 0.1 M HCl(aq), (b) 0.1 M NaCl(aq), (c) 0.1 M LiCl(aq), and (d) 0.1 M TPACl(aq). Correlation between the hydrated size for the corresponding cations in (a–d) with the (e) intercalation peak current density, *j*_peak_ and (f) total charge passed density, *Q*_peak_.

Additional cyclic voltammetric measurements were performed specifically with (Sb_2_Te_3_)_2_(Sb_2_)_2_ in electrolytes with the same anion but with cations that differed in hydrated size (H^+^, Li^+^, Na^+^, and tetrapropylammonium (TPA^+^)). Fig. 3a–d present these data, where the blue trace in each panel corresponds to a control response recorded with a glassy carbon electrode. The (Sb_2_Te_3_)_2_(Sb_2_)_2_ ingot electrodes exhibited some form of a pre-wave prior to the onset of H_2_(g) evolution in each electrolyte, but the magnitude and peak position varied. For H^+^, the cathodic peak current was 2.44 mA cm^−2^ at −0.35 V, with a very narrow wave shape. For Na^+^, the peak occurred at a different absolute potential (−1.01 V) and a lower peak current magnitude of 1.4 mA cm^−2^ and a broader shape. For Li^+^, the peak current was observed at −1.06 V and a peak current magnitude of 1.34 mA cm^−2^. For TPA^+^, the pre-wave peak current was 0.62 mA cm^−2^ at −1.09 V. For all electrolytes, at potentials equal to or more negative than the pre-wave, material was observed streaming from the electrode into solution. However, the amount of material streaming in HCl(aq) was noticeably less, with more material ejected as large flakes and much more vigorous evolution of H_2_(g) bubbles. Fig. 3e and f present the peak currents and integrated charge passed, respectively, from these voltammetry as a function of the hydrated radius size. The hydrated radii for H^+^, Li^+^, Na^+^, and TPA^+^ are 2.82, 3.58, 3.82, and 4.52 Å, respectively.^21^ A clear linear correlation was observed between the peak current and hydrated radius, with lowered peak currents for larger cations. The integrated charge passed exhibited a weaker linear correlation with cation size. A correlation between the peak currents and water coordination number for each cation^22–25^ was also noted (SI, Fig. S2).

**Fig. 4:**
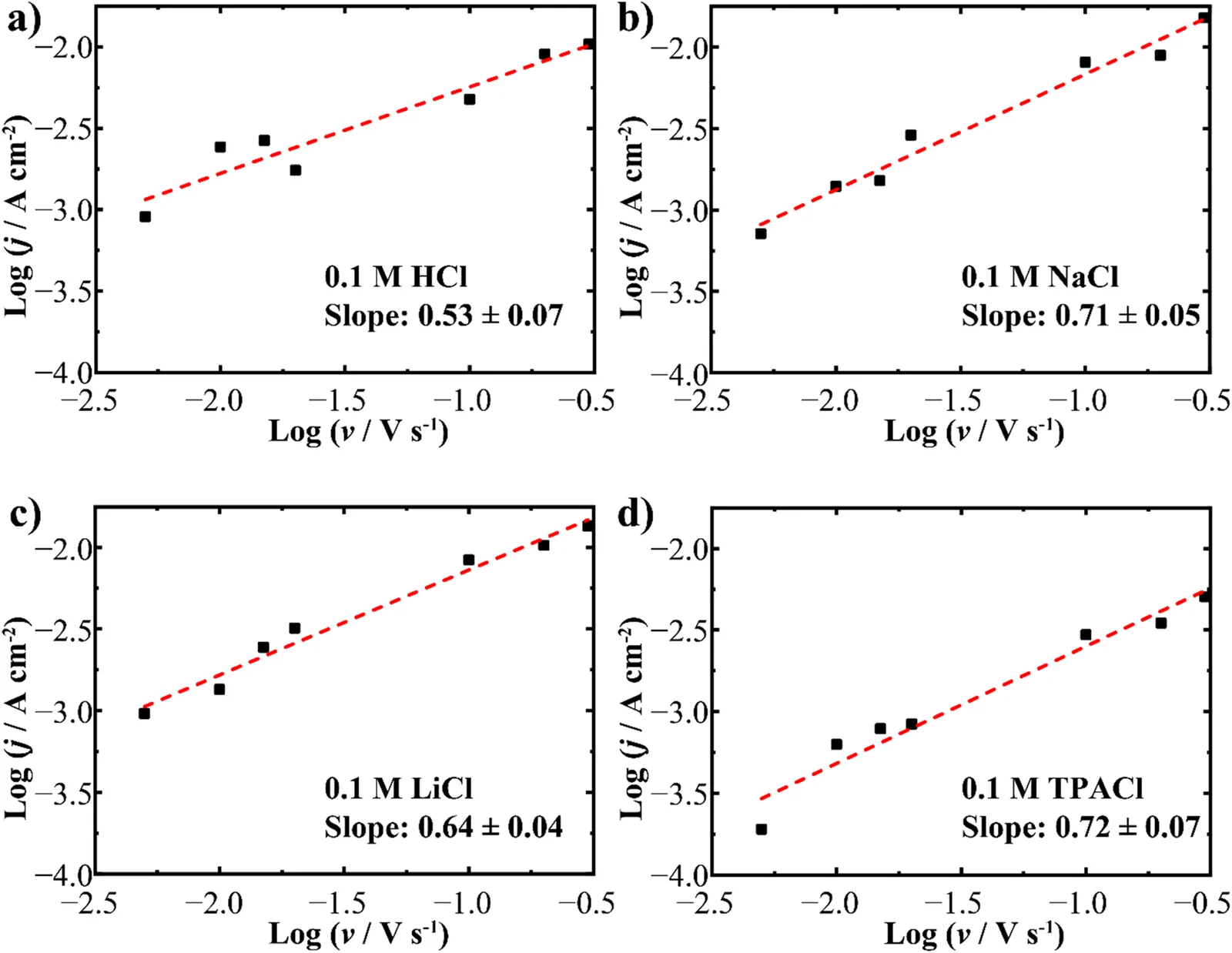
Plots of the peak current densities *vs.* scan rate (5 mV s^−1^ to 300 mV s^−1^) on a log–log scale for (a) 0.1 M HCl(aq), (b) 0.1 M NaCl(aq), (c) 0.1 M LiCl(aq), and (d) 0.1 M TPACl(aq).

To identify whether the pre-wave feature was diffusive or surface-bound in nature, additional cyclic voltammetric measurements were collected as a function of scan rate (Fig. 4). In each panel, the peak current density *vs.* scan rate data are presented in log–log format to determine the power-law relationship,^26^

1 *i*_peak_ = *av*^*b*^

where *a* is a proportionality term that collects the defining set of physical constants of the measurement and *b* is the order of dependence on the scan rate. For a purely diffusive redox process, *b* = 0.5 while for a strictly surface-confined redox reaction, *b* = 1.^26^ The measured *b* values were 0.53 ± 0.07, 0.71 ± 0.05, 0.64 ± 0.04, and 0.72 ± 0.07 for H^+^, Li^+^, Na^+^, and TPA^+^, respectively.

**Fig. 5:**
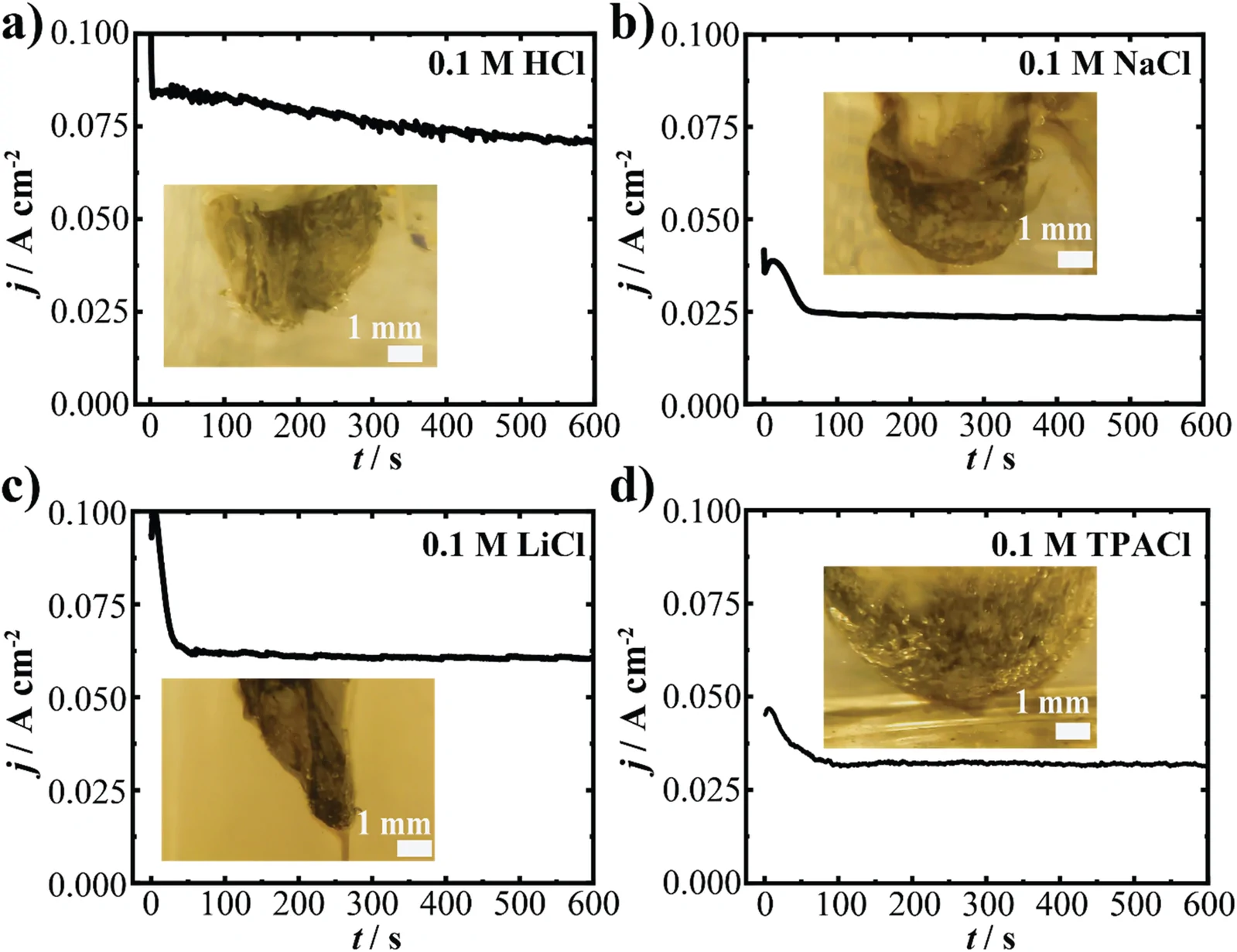
Chronoamperometric (current *vs.* time) responses recorded for (Sb_2_Te_3_)_2_(Sb_2_)_2_ electrodes held at *T* = 25 °C and *E* = −2.0 V *vs.* Ag/AgCl in (a) 0.1 M HCl(aq), (b) 0.1 M NaCl(aq), (c) 0.1 M LiCl(aq), and (d) 0.1 M TPACl(aq). Insets: an optical photograph of the streaming of materials off the electrode surface during each experiment.

To facilitate collection of material streaming from the electrode into solution, separate chronoamperometry experiments were performed. When the potential was held at a potential just passed the pre-waves seen in Fig. 3, material streamed off the electrode for only 10–60 s before cessation. Several different applied potentials were explored, with −2.0 V considered optimal for the following two reasons. First, this potential was more negative than the Sb^3+^ reduction peak. Second, this was the least negative potential where a continuous stream of delaminated materials was consistently observed for long (10 minutes) durations. Potentials less negative than −2.0 V tended to result exfoliation that stopped after ∼1 min. Fig. 5 illustrates the results when −2 V was applied to (Sb_2_Te_3_)_2_(Sb_2_)_2_ electrodes for 10 min. At this potential, copious amounts of material streamed off the electrode during the entire time period, facilitating subsequent collection of ejected materials. The insets in Fig. 5 illustrate the quantity of material streaming off the ingot electrodes at this applied potential. The current–time transients varied somewhat in magnitude but had three principal and consistent features. First, the current density was largest at the start of the experiment and decayed rapidly within the first 60–100 s. Following this fast decay, the current remained relatively steady, with some periodic noise oscillations. After this decay, the stream of delaminated material slowed. Second, neither the initial nor long time periods were well described by instantaneous or progressive nucleation models typically observed during electrodeposition reactions.^27^ Additionally, there was also a noticeable difference in size of H_2_(g) bubbles at the ingot electrode/water interface. TPA^+^ electrolyte led to visually larger bubbles than when using either Li^+^ or Na^+^ electrolytes. Third, the mass yields were not equivalent for the different cations. For experiments lasting 30 min, approximately 1 mg of material was collected with H^+^, Li^+^, and TPA^+^. In comparison, approximately 25 mg were obtained with Na^+^. Additional experiments were performed to assess the impact of electrolyte concentration. In experiments lasting 10 min and using NaCl electrolytes with concentrations of either 0.001, 0.01, 0.1, or 1.0 M, the masses of collected materials were 0.550, 1.967, 3.961, and 15.301 mg, respectively.

Separate measurements were performed to clarify the effect of pH of the bulk solution. The LiCl, NaCl, and TPACl electrolytes were unbuffered with pH = 6.8 ± 0.5. Simple immersion of the ingot electrodes into these electrolytes without electrochemical bias resulted in no perceptible change to the material surface or chemical dissolution of the bulk material. Since the pH of the HCl electrolyte was much lower (*i.e.* 1), the possibility of etching was examined in greater detail. Scanning electron microscopy was performed on a shaving of the bulk ingot before and after immersion into 0.1 M HCl(aq) for 1 h without any applied electrochemical bias. If appreciable chemical etching/dissolution occurred, the expectation was that the shaving size/volume would noticeably decrease. The observation was that the shaving was unchanged after immersion, with even the terrace domain sizes being unaffected by HCl (SI, Fig. S3). Hence, acid etching was not inferred as relevant. Additionally, although the total currents passed were high in 0.1 M HCl(aq), the fact that much less mass was collected as compared to experiments performed with NaCl suggested that H_2_(g) evolution did not particularly enhance H^+^ intercalation. This point has been previously argued for cathodic exfoliation of graphite in water.^28^

### Exfoliated material characterization

Representative scanning electron microscopy and atomic force microscopy data for as-cast electrochemical exfoliated materials are presented in the (SI, Fig. S4 and S5). Lateral dimensions of 100 representative exfoliated structures were measured. The lateral length of materials prepared with either TPA^+^, Na^+^, Li^+^, or H^+^ were 8.9 ± 6.8 µm, 5.1 ± 4.3 µm, 6.8 ± 7.3 µm, and 3.4 ± 5.4 µm, respectively. The thinnest exfoliated materials using Li^+^ were 4.5 nm thick, which was equivalent to 6 layers of (Sb_2_Te_3_)_2_(Sb_2_)_2_.

**Fig. 6:**
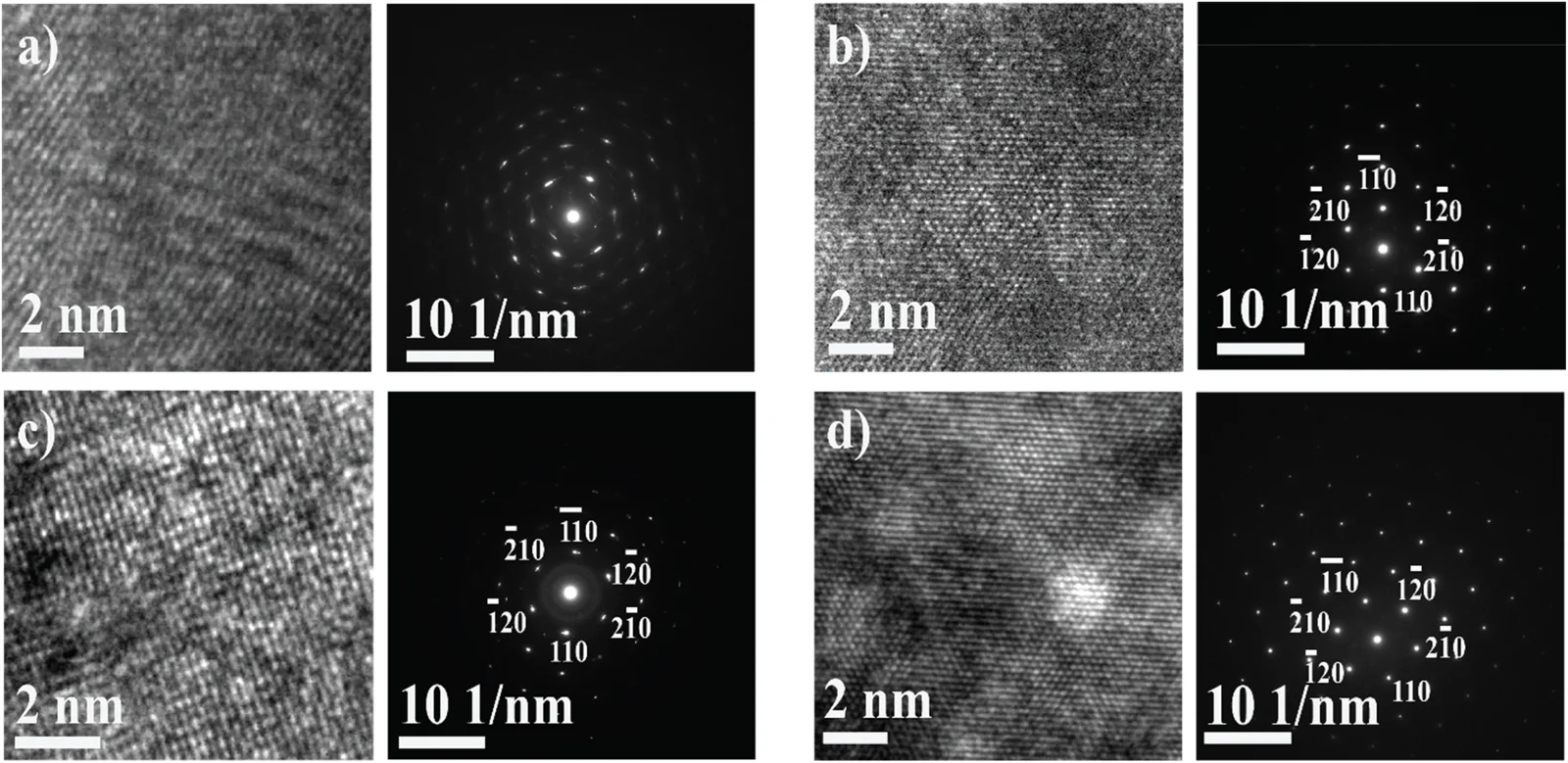
High resolution transmission electron micrographs (HR-TEM) and corresponding selected area diffraction of exfoliated flakes collected from electrochemical exfoliation of (Sb_2_Te_3_)_2_(Sb_2_)_2_ using (a) H^+^, (b) Na^+^, (c) Li^+^, and (d) TPA^+^ containing electrolytes.

Bright-field transmission electron microscopy of similar materials cast onto Cu grid substrates was separately performed. Fig. 6 shows a compilation of high-resolution transmission electron micrographs and corresponding selected area electron diffraction (SAED) patterns. Fig. 6a shows representative data for material collected using strong HCl acid as the electrolyte. In these materials, lattice fringes were apparent, with average *d* spacing of 0.36 nm for (101) crystallographic plane. The crystalline domains were continuous only over short distances (<10 nm). This point was further emphasized in the corresponding SAED data. Four separate samples were examined and yielded the nominally equivalent SAED data. The pattern exhibited azimuth elongated streaks rather than discrete spots, indicative of multiple crystalline domains within the interrogated volume. Azimuth elongation was due to a distribution of orientations of crystallites, *i.e.* not all crystallites were perfectly aligned in the *c*-axis. Therefore, the pattern contained features that were suggestive of, but could not be indexed cleanly to, the ordered structure of (Sb_2_Te_3_)_2_(Sb_2_)_2_.

Fig. 6b shows data collected with Na^+^ electrolyte. These materials more readily showed lattice fringes consistent with the 0.21 nm *d* spacing for (110) planes of the bulk (Sb_2_Te_3_)_2_(Sb_2_)_2_ structure. SAED measurements were performed on 5 distinct materials that yielded the same SAED data features. These SAED data could be unambiguously indexed to a 6-fold symmetric (Sb_2_Te_3_)_2_(Sb_2_)_2_ single crystal. Fig. 6d shows representative results for material prepared with TPA^+^. SAED data were collected from 5 flakes and yielded indistinguishable SAED patterns. These data confirmed the single-crystalline nature observed in the high-resolution TEM images along the [001] direction (*c*-axis) of the (Sb_2_Te_3_)_2_(Sb_2_)_2_ structure. The measured *d*-spacing again matched the (110) plane. The SAED also contained faint additional superlattice spots arranged in a hexagonal pattern around each primary Bragg reflection. These features were consistent with kinematically forbidden reflections of (100) associated with the stacking sequence in the *R*3̄*m* structure. The appearance of those spots denoted the broken symmetry along the *c*-axis, which leads to dynamic scattering, or a superlattice (√3*a* × √3*a*, *R* = 30°) in the *a*–*b* plane while maintaining the stacking order in the *c*-axis. To examine whether the appearance of forbidden reflections are due to dynamic scattering, the sample was slightly tilted off of the [001] zone axis. After tilting, the forbidden reflection spot remained, indicating this was not dynamic scattering effect (SI, Fig. S6).

Fig. 6c shows the corresponding data for material collected with Li^+^. High resolution TEM indicated multiple small domains. The measured *d*-spacing of 0.21 nm again matched with the value expected for the (110) plane. The SAED data showed 6-fold symmetry, generally consistent with the structure of (Sb_2_Te_3_)_2_(Sb_2_)_2_. However, each spot was blurred about the center, suggesting multiple crystals within the probed volume. Unlike any other samples, a small fraction (2 out of 5 flakes) exhibited decidedly distinct diffraction data (SI, Fig. S7). These SAED data did not possess 6-fold symmetry and were instead suggestive of the presence of LiTe_3_ and LiSbTe_2_, *i.e.* products from an electrochemically-driven reaction with Li^+^.

**Fig. 7:**
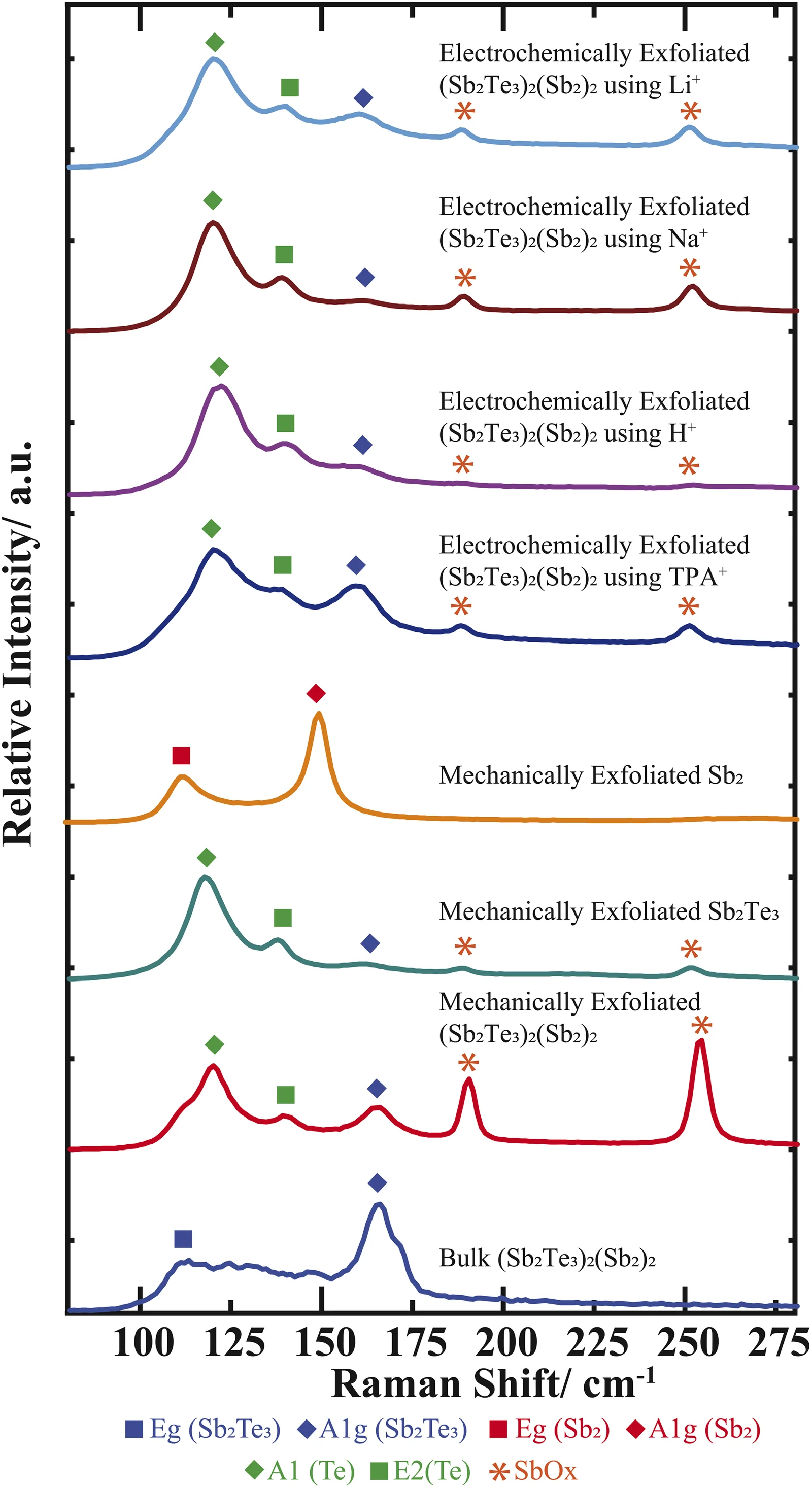
Raman spectra of a polycrystalline (Sb_2_Te_3_)_2_(Sb_2_)_2_ ingot, mechanically exfoliated (Sb_2_Te_3_)_2_(Sb_2_)_2_, mechanically exfoliated Sb_2_Te_3_, mechanically exfoliated Sb_2_, and electrochemically exfoliated (Sb_2_Te_3_)_2_(Sb_2_)_2_ using either Li^+^, Na^+^, H^+^ and TPA^+^ containing electrolyte.

Raman spectroscopy was also employed to characterize the materials obtained with various cations. For context, Fig. 7 presents Raman spectra for related reference materials measured dry in air: bulk (Sb_2_Te_3_)_2_(Sb_2_)_2_, mechanically exfoliated (Sb_2_Te_3_)_2_(Sb_2_)_2_, mechanically exfoliated Sb_2_Te_3_, and mechanically exfoliated Sb_2_. The phonon mode assignments are denoted by the colored symbols above each spectral feature. Each of these materials exhibited similar but distinguishable features in the range of 75–200 cm^−1^. Notably, the Raman spectra for bulk Sb_2_Te_3_ and (Sb_2_Te_3_)_2_(Sb_2_)_2_ strongly resembled each other, with the A1g^29^ mode being the most prominent for both materials. In contrast, mechanical exfoliation of both materials severely attenuated the A1g mode and introduced two phonon modes (A1 and E2)^30^ for elemental Te. In addition, two other features at 190 cm^−1^ and 253 cm^−1^ appeared that were consistent with SbO_*x*_.^31^ For comparison, the spectra for bulk Sb_2_ (data not shown) and mechanically exfoliated Sb_2_ were essentially indistinguishable, featuring only the Eg and A1g modes at 112 and 150 cm^−1^, respectively, of Sb_2_.^31^ No phonon modes for SbO_*x*_ were observed even at the maximum incident laser intensity.

The Raman spectra for the material collected from the (Sb_2_Te_3_)_2_(Sb_2_)_2_ electrodes in Li^+^ electrolyte showed several interesting features. First, none of the materials exhibited spectra that could be ascribed to LiTe_3_ and LiSbTe_2_, in agreement with the prior reports that these materials have no intense Raman active modes in this spectral range.^32,33^ Second, two sub-populations of samples were observed. A minority set of flakes yielded Raman spectra in close agreement with Sb_2_, although the Eg and A1g modes were blue and red shifted, *i.e.* had less spacing than seen with pure Sb_2_ materials. The majority of these interrogated materials showed Raman spectra very similar to mechanically exfoliated (Sb_2_Te_3_)_2_(Sb_2_)_2_, albeit with much less intense features for SbO_*x*_ at the same incident laser intensity. These spectra were essentially identical in form to spectra collected from material prepared with TPA^+^, Na^+^, and H^+^. However, the position of the A1g mode in these spectra varied amongst the cations. For these electrochemically exfoliated samples, the time-dependence of the intensity of the peaks associated with SbO_*x*_ was examined. Raman spectra were obtained for freshly exfoliated samples and samples that had been exfoliated and then stored in air for approximately 12 months (SI, Fig. S8). There was no significant difference in the intensity of SbO_*x*_ peaks. Hence, the sum Raman and electron diffraction data indicated electrochemically exfoliated materials were chemically stable in air for storage, washing, and during sonication.

## Discussion

The presented data collectively speak to three points. First, cation intercalation into (Sb_2_Te_3_)_2_(Sb_2_)_2_ can occur at negative bias and cause exfoliation of layered sections. Second, electrochemical exfoliation of the (Sb_2_Te_3_)_2_(Sb_2_)_2_ structure is milder and less chemically damaging than mechanical exfoliation. Third, cathodic electrochemical intercalation and exfoliation of (Sb_2_Te_3_)_2_(Sb_2_)_2_ is influenced by the size of the cation. These points are discussed individually below.

### Electrochemical intercalation and exfoliation

The presented data are clear that (Sb_2_Te_3_)_2_(Sb_2_)_2_ has redox activity prior to the onset of H_2_(g) evolution in aqueous electrolytes. Based on the similarity of the voltammetric data for Sb_2_Te_3_ and Sb_2_ electrodes and with the prior electrochemical literature on Sb,^18,34^ a reasonable conclusion is this redox activity is associated with reduction of oxidized Sb species. This point was further corroborated by X-ray photoelectron spectra obtained on bulk (Sb_2_Te_3_)_2_(Sb_2_)_2_ before and after application of −2.0 V (SI, Fig. S9). For bulk Sb_2_, the possibility of surface oxides is reasonable. For Sb_2_Te_3_ and (Sb_2_Te_3_)_2_(Sb_2_)_2_, Sb nominally in the +3 oxidation state is present (the Sb oxidation state is 0 in a Sb_2_ bilayer). From the observation of visible but short-lived streaming of material at potentials prior to H_2_(g) evolution, the magnitude of the integrated charge, and that at more extreme bias an abundance of material was displaced from the surface of the ingot electrode, the data indicates that electrochemical cation intercalation consistently occurs in (Sb_2_Te_3_)_2_(Sb_2_)_2_ following the reduction of Sb species.

One possible consequence of reduction of Sb within the quintuple Sb_2_Te_3_ layers in (Sb_2_Te_3_)_2_(Sb_2_)_2_ is total destruction of the material. That is, all Sb sites are reduced and the sub-lattice of quintuple layers are destroyed. The presented data are not consistent with this possibility. The morphology of the exfoliated materials imaged by SEM and AFM are consistent with thin flake with large basal planes. Separately, the electron diffraction data (Sb_2_Te_3_)_2_(Sb_2_)_2_ exfoliated electrochemically with the two largest cations, Na^+^ and TPA^+^, showed the presence of kinematically forbidden reflections. Since these reflections are not due to dynamic scattering, a possible explanation is that intercalated guest species occupy interstitial positions within the van der Waals gaps of the native structure of (Sb_2_Te_3_)_2_(Sb_2_)_2_. Such superlattice structures imply that the base crystal structure is preserved, rather than destroyed, by intercalation. However, for the smaller Li^+^ cation, the formation of new phases (*i.e.* LiTe_3_ and LiSbTe_2_) were observed, consistent with at least some chemical attack in Sb_2_Te_3_ layer by the intercalated ion. Still, even for the Li^+^, the majority of interrogated materials yielded electron diffraction satellite peaks indicative of domains that were slightly rotated from each other rather than completely chemically decompose.^35^ Rather, the observation of lithiated structures of (Sb_2_Te_3_)_2_(Sb_2_)_2_ further supports the premise that Li^+^ reaches the sub-surface of the material by transport between layers, *i.e.* intercalates.

### Mild electrochemical exfoliation

A clear take-home point from the Raman spectra is that electrochemical exfoliation does not produce flakes equivalent to the material obtained by mechanical exfoliation. Under the laser irradiation used for Raman spectroscopy, the decomposition of mechanically exfoliated Sb_2_Te_3_ and (Sb_2_Te_3_)_2_(Sb_2_)_2_ into SbO_*x*_ was evident. Conversely, little to no oxidation was observed in the electrochemically exfoliated materials.

The electrochemically exfoliated materials were consistent with multiple sites for ion intercalation. For example, if *m* = *n* = 1 in the stacking structure of (Sb_2_Te_3_)_*m*_(Sb_2_)_*n*_, only a single type of gap between Sb_2_Te_3_ and Sb_2_ would be expected with a spacing of 3.1 Å.^5,36^ Ion insertion and resultant exfoliation would occur at the same plane as mechanical exfoliation and so the obtained materials would be expected to be similar. However, this was not observed experimentally. Under the laser irradiation used for Raman spectroscopy, the decomposition of mechanically exfoliated (Sb_2_Te_3_)_2_(Sb_2_)_2_ into SbO_*x*_ was evident. Conversely, little to no oxidation was observed in the electrochemically exfoliated materials.

None of the cations used here can ‘fit’ within any of the gaps if fully solvated. Hence, ion intercalation clearly requires at least partial desolvation prior to insertion.^37^ Although no measurements were performed that directly probed this step, it seems plausible that such a chemical step could contribute to the larger *b* values than predicted for simple redox activity of purely surface-confined species observed in the scan rate-dependent data. Still, not all of the interlayer spaces should be accessible to all desolvated cations. Desolvated Li^+^, Na^+^, and H^+^ are sufficiently small (0.67, 0.95, and 1.38 Å, respectively)^21,38^ that they could readily fit within all of the van der Waals gaps. However, a preference towards insertion in the largest gap between adjacent Sb_2_Te_3_ quintuple layers is statistically expected from a kinetic perspective since the largest gap should elicit the lowest kinetic barrier for the rate of ion intercalation rate.^39^ Predominate ion insertion within the Sb_2_Te_3_/Sb_2_Te_3_ gap would cleave the material and expose Te-terminated Sb_2_Te_3_ layers. As observed in the Raman spectra for mechanically cleaved Sb_2_Te_3_ (which can only expose these same surface planes), this type of exfoliation does not result in extensive formation of SbO_*x*_. Hence, the electrochemical exfoliation of (Sb_2_Te_3_)_2_(Sb_2_)_2_ appears to be selective towards non-destructive exfoliation.

Since mechanical exfoliation of (Sb_2_Te_3_)_2_(Sb_2_)_2_ clearly resulted in a large amount of SbO_*x*_, we infer that such exfoliation allows cleavage across each type of layer gap with similar probabilities. Based on the presented data, cleavage along some of these layer gaps results in material that are less chemically stable.

The data argue that cleavage along the Sb_2_Te_3_/Sb_2_ gap is likely not the most chemically destructive to (Sb_2_Te_3_)_2_(Sb_2_)_2_. This type of exfoliation would result in a Te-terminated Sb_2_Te_3_ plane and an Sb_2_ sheet supported by an underlayer of Sb_2_. As mentioned above, the Te-terminated Sb_2_Te_3_ does not appear particularly susceptible to oxidation. Similarly, the Raman spectra for mechanically exfoliated Sb_2_, which only cleaves along the surface plane separating Sb_2_ layers, indicate that basal plane Sb_2_ does not readily oxidize in air. Rather, the data implicate exfoliation along the gap between Sb_2_/Sb_2_ layers as particularly chemically damaging.

**Fig. 8:**
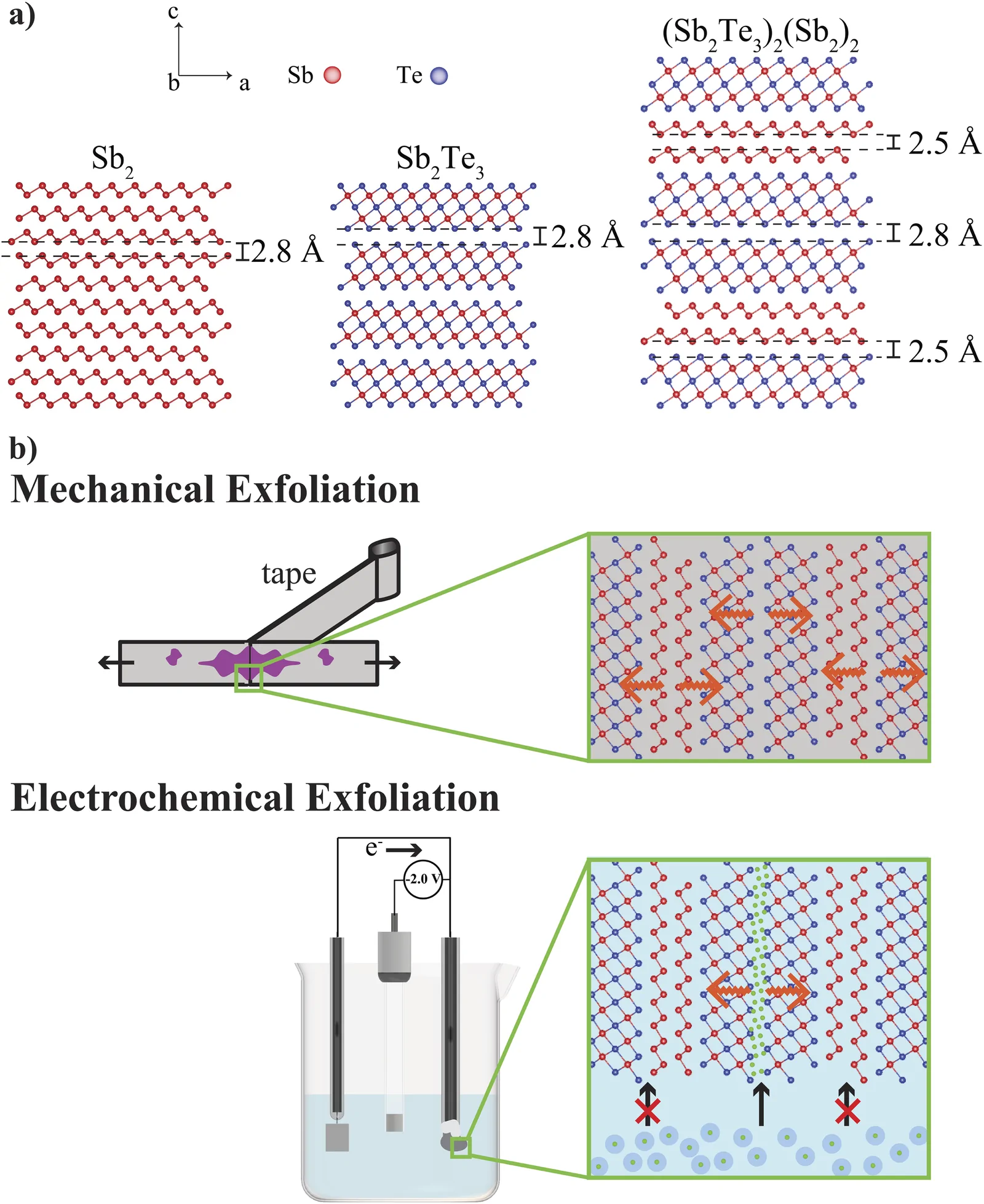
(a) Schematic diagram of stacking in (Sb_2_Te_3_)_2_(Sb_2_)_2_, Sb_2_, and Sb_2_Te_3_ and the corresponding interlayer spacing between Sb_2_Te_3_/Sb_2_Te_3_, Sb_2_Te_3_/Sb_2_, Sb_2_/Sb_2_ in (Sb_2_Te_3_)_2_(Sb_2_)_2_, adjacent Sb_2_ bilayer in Sb_2_, and adjacent Sb_2_Te_3_ quintuple layers in Sb_2_Te_3_. (b) Schematic depiction of (top) mechanical exfoliation of (Sb_2_Te_3_)_2_(Sb_2_)_2_ by the tape method and (bottom) cathodic electrochemical exfoliation of (Sb_2_Te_3_)_2_(Sb_2_)_2_. The jagged orange arrows indicate the direction of tensile stress from either mechanical pulling or ion insertion.

The Sb_2_/Sb_2_ gap in (Sb_2_Te_3_)_2_(Sb_2_)_2_ is notable because it is significantly smaller than the Sb_2_/Sb_2_ gap in stacks of Sb_2_ (*i.e.* bulk antimonene, Fig. 8a). In antimonene crystals, the inter-layer spacing is 2.8 Å.^40–42^ The large gap in antimonene is consistent with pure London dispersion interactions between layers, as antimonene has been described as a close analog of graphene.^43^ The noticeably smaller Sb_2_/Sb_2_ gap in (Sb_2_Te_3_)_2_(Sb_2_)_2_ reflects some interaction with the adjacent Te-terminated Sb_2_Te_3_ quintuple layer. Covalent bonding between these two layers has repeatedly been ruled out by multiple groups.^2,3,44^ However, the heterointerface van der Waals between Sb_2_ and Sb_2_Te_3_ is not a true van der Waals, as it is subjected to short-range orbital overlap at the Te edge of Sb_2_Te_3_ and Sb of Sb_2_. Specifically, due to partially delocalized, highly aligned p-orbitals between Sb and Te, the Sb–Te bond in Sb_2_Te_3_ is metavalent, *i.e.* high polarizability.^45^ Electron density does not abruptly end at the Te edge of Sb_2_Te_3_ but can extend across the van der Waals gap, allowing weak hybridization with Sb of Sb_2_.^46^ Such hybridization with an adsorbed Sb adlayer from metavalent bonding has been observed with Bi_2_Se_3_.^42^

If (Sb_2_Te_3_)_2_(Sb_2_)_2_ is cleaved along the Sb_2_/Sb_2_ gap, two single Sb_2_ layers supported by the Te-terminated Sb_2_Te_3_ quintuple layer are produced. We posit that the heteropolar interaction between Sb_2_ and the Te-terminated Sb_2_Te_3_ quintuple layer greatly enhances the susceptibility of Sb_2_ towards oxidation in air and in water. That is, the exposed Sb_2_ layers from this cleavage are explicitly not chemically equivalent to exfoliated antimonene. Accordingly, exfoliation of (Sb_2_Te_3_)_2_(Sb_2_)_2_ in this manner should result in different resultant materials. We interpret the results presented here as consistent with this premise and indicative that electrochemical exfoliation is a milder method to cleave (Sb_2_Te_3_)_2_(Sb_2_)_2_ than mechanical exfoliation (Fig. 8b).

### Role of cation size

This work also clearly illustrates that electrochemical intercalation and exfoliation of (Sb_2_Te_3_)_2_(Sb_2_)_2_ is sensitive to the specific cation identity. Although electrochemical exfoliation yielded much less SbO_*x*_ content, the Raman spectra were not equivalent across the four cations.

Exfoliation performed with TPA^+^ electrolytes visibly elicited the least amount of streaming material and smallest peak current density. Additionally, slightly less production (relative to the materials prepared with the other cations) of SbO_*x*_ was observed with TPA^+^, consistent with the premise that even if desolvated, it is too large to intercalate consistently within the smaller Sb_2_/Sb_2_ gap. Accordingly, large cations like TPA^+^ do not efficiently exfoliate (Sb_2_Te_3_)_2_(Sb_2_)_2_ but also are mild intercalants.

The slightly smaller but more easily desolvated (SI, Fig. S2) Na^+^ cations definitely yielded more exfoliated material relative to experiments performed with the other cations but did not produce much more intense signatures of SbO_*x*_ in the Raman spectra. We interpret this to mean that statistically, on average, Na^+^ was able to insert into the (Sb_2_Te_3_)_2_(Sb_2_)_2_ electrode but did not predominately intercalate within the Sb_2_/Sb_2_ gap. Additionally, the lack of diffraction signatures for any sodiated phases suggest that Na^+^ did not chemically react with, or largely perturb the structure of, (Sb_2_Te_3_)_2_(Sb_2_)_2_ upon insertion. Accordingly, Na^+^ represents a more efficient but still gentle cation for exfoliation of (Sb_2_Te_3_)_2_(Sb_2_)_2_.

In contrast, H^+^ yielded unambiguous evidence of extreme chemical reaction with (Sb_2_Te_3_)_2_(Sb_2_)_2_ during exfoliation. The electron microscopy and diffraction data made clear that H^+^ insertion definitively does perturb the (Sb_2_Te_3_)_2_(Sb_2_)_2_ structure. Curiously, the Raman spectra for material produced by exfoliation with H^+^ yielded the least intense features for SbO_*x*_ but that should not be interpreted as evidence for mild exfoliation. Rather, the acidic conditions used for exfoliation also facilitated rapid dissolution of SbO_*x*_ species,^47^ leaving behind only the materials generated by exfoliation along the larger layer gaps. Additionally, the concomitant generation of H_2_(g) lowered the overall efficiency for exfoliation, resulting in less material collected than in experiments with Na^+^. Accordingly, H^+^ does not have desirable characteristics as an ion intercalant explicitly for exfoliation.

Finally, Li^+^ represented an intermediate case. As with Na^+^, ample material streamed off the electrode during the electrochemical exfoliation step. These materials yielded some but not excessive amounts of SbO_*x*_, indicating that Li^+^ does not seem to exclusively intercalate within the most sensitive gaps within the (Sb_2_Te_3_)_2_(Sb_2_)_2_ structure. However, unlike Na^+^, lithiated phases were detected, indicating that this type of ion intercalation resulted in some undesired chemistry with (Sb_2_Te_3_)_2_(Sb_2_)_2_. It is not clear whether lithiated compound formation occurred concurrently with cleavage along the Sb_2_/Sb_2_ gap or was a consequence of Li^+^ insertion within any gap. Since only a minor fraction of lithiated materials were detected and since the majority of measurements yielded structures consistent with (Sb_2_Te_3_)_2_(Sb_2_)_2_, it seems that lithiation is not the predominant effect of Li^+^ insertion. In this regard, exfoliation *via* Li^+^ insertion may still prove fruitful for realizing pristine 2D materials from (Sb_2_Te_3_)_2_(Sb_2_)_2_.

## Conclusions

This work presents electrochemical intercalation and exfoliation of (Sb_2_Te_3_)_2_(Sb_2_)_2_ using several cations: H^+^, Na^+^, Li^+^, and TPA^+^. The data reveals that cathodic electrochemical intercalation and exfoliation of (Sb_2_Te_3_)_2_(Sb_2_)_2_ occur consistently after Sb^3+^ reduction. The exfoliated materials are flat flakes that retain their crystallinity to varying degrees. Unlike mechanically exfoliated (Sb_2_Te_3_)_2_(Sb_2_)_2_, electrochemically exfoliated (Sb_2_Te_3_)_2_(Sb_2_)_2_ is noticeably less prone to degradation that produces SbO_*x*_. Cations appear to preferentially intercalate between the largest gap of adjacent quintuple Sb_2_Te_3_ layers, while mechanical exfoliation results in a less selective layer separation, which potentially takes place at all three gap types within the AA′B′ABB′C′BCC′A′C structure. The cumulative results implicate particularly damaging exfoliation if performed across the Sb_2_/Sb_2_ gap. This work thus provides some insight on exfoliation of (Sb_2_Te_3_)_*m*_(Sb_2_)_*n*_ with *m* = *n*, to yield pristine few or single layer 2D materials. Work is ongoing to identify electrochemical parameters that efficiently yield a single unit of quintuple Sb_2_Te_3_ and bilayer Sb_2_, which requires concomitant intercalation of adjacent Sb_2_/Sb_2_ and Sb_2_Te_3_/Sb_2_Te_3_ gap.

## Conflicts of interest

There are no conflicts to declare.

## Supplementary Material

RA-016-D6RA02757A-s001

## Data Availability

All of the data presented in this article, including diffraction patterns, electron micrographs, cyclic voltammograms, chronoamperograms, Raman spectra, and atomic force micrographs are listed under “Electrochemical Intercalation and Exfoliation of Sb_4_Te_3_” and available at Deep Blue Data at https://deepblue.lib.umich.edu/data/concern/data_sets/m900nv79v. Supplementary information (SI) is available. See DOI: https://doi.org/10.1039/d6ra02757a.
