# Supplementary material for: Electrochemical intercalation and exfoliation of (Sb2Te3)2(Sb2)2
Source: RSC Adv. 2026 Sep 1;16(42):51204–16. doi: 10.1039/d6ra02757a (PMC13531041; doi:10.1039/d6ra02757a)
Supplement: Supplementary file 1 [file RA-016-D6RA02757A-s001.pdf]

Supplementary Information (SI) for RSC Advances.
This journal is © The Royal Society of Chemistry 2026

Electrochemical Intercalation and Exfoliation of $(Sb_2Te_3)_2(Sb_2)_2$

Tung T. Nguyen[1], Yimo Hou,[3] Selene Koremenos - Tsebelis,[3] Nishkarsh Agarwal,[3] Robert Hovden,[2,3] Pierre Ferdinand P. Poudeu,[3] Stephen Maldonado[1,2]*

1. *Department of Chemistry, University of Michigan, Ann Arbor, Michigan 48109-1055, USA*

2. *Program in Applied Physics, University of Michigan, Ann Arbor, Michigan 48109-1055, USA*

3. *Material Science and Engineering, University of Michigan, Ann Arbor, MI, 48109, USA*

*smald@umich.edu

Section S1. Contents

This document contains supporting information for the manuscript entitled *"Electrochemical Intercalation and Exfoliation of $(Sb_2Te_3)_2(Sb_2)_2$"*. Section S2 presents X-ray diffraction data for as-synthesized Sb_2 and Sb_2Te_3 with the corresponding references of powder XRD for Sb_2 and Sb_2Te_3. Section S3 indicates the correlation between J_{peak} and coordination number for various cations. Section S4 represents scanning electron micrographs of exfoliated flakes submerged in 0.1 M HCl. Section S5 presents atomic force microscopy data of thin exfoliated $(Sb_2Te_3)_2(Sb_2)_2$ using Li^+ and thickness distribution of flakes exfoliated using various cations including TPA^+, Li^+, Na^+, and H_3O^+. Section S6 presents scanning electron micrographs of exfoliated $(Sb_2Te_3)_2(Sb_2)_2$ using various cations cast on Si wafer. Section S7 presents electron diffraction of exfoliated $(Sb_2Te_3)_2(Sb_2)_2$ using TPA^+ which was obtained at slightly tilted off [001] zone axis with the corresponding gray value profile plot. Section S8 presents selected area electron diffraction (SAED) of byproducts of $(Sb_2Te_3)_2(Sb_2)_2$ in 0.1 M LiCl*(aq)* at -2.0 V vs. *E*(Ag/AgCl). Section S9 presents Raman spectra of exfoliated flakes which was stored in air for approximately 12 months. Section S10 presents X-ray photoelectron spectra of $(Sb_2Te_3)_2(Sb_2)_2$ before and after application of -2.0 V vs. *E*(Ag/AgCl) bias. Section S11 represents elemental mapping of electrochemically exfoliated $(Sb_2Te_3)_2(Sb_2)_2$ in 0.1 M NaCl. Section S12 lists the works cited.

Section S2. X-ray Diffraction of Sb_2 and Sb_2Te_3

Figure S1 depicts the consistency between the XRD data of Sb_2 and Sb_2Te_3 with the corresponding reference obtained from Inorganic Crystal Structure Database. As synthesized Sb_2 exhibited (0,0,3) at 23.7º, (0,1,2) at 28.7º, (1,0,4) at 40.1º, (1,1,0) at 41.9º, (0,0,6) at 48.4º, (2,0,2) at 51.6º, (0,2,4) at 59.4º, (1,2,2) at 68.6º, (2,1,4) at 75.4º. Observed Braggs diffraction peaks of as-synthesized Sb_2Te_3 were (0,0,9) at 26.3º, (0,1,5) at 28.2º, (1,0,10) at 38.3º, (1,1,0) at 42.4º, (0,0,15) at 44.6º, (2,0,5) at 51.7º, (0,2,10) at 58.5º, (1,2,5) at 68.9º, (2,1,0) at 74.7º. While the Bragg diffraction peaks of as-synthesized Sb_2Te_3 and $(Sb_2Te_3)_2(Sb_2)_2$ (Figure 1, Figure S1) were quite similar, a key difference between the two structures were the c-plane peaks such as (0,0,9) for Sb_2Te_3 at 26.3º, which corresponded to c parameter of 30.46 Å, and (0,0,24) for $(Sb_2Te_3)_2(Sb_2)_2$ at 25.5º, which corresponded to longer c-parameter of 83.46 Å due to inclusion of Sb_2 in the unit cell. Overall, the XRD of the as-synthesized powder matches well with the reference, which confirms the identity and composition of each ingot.

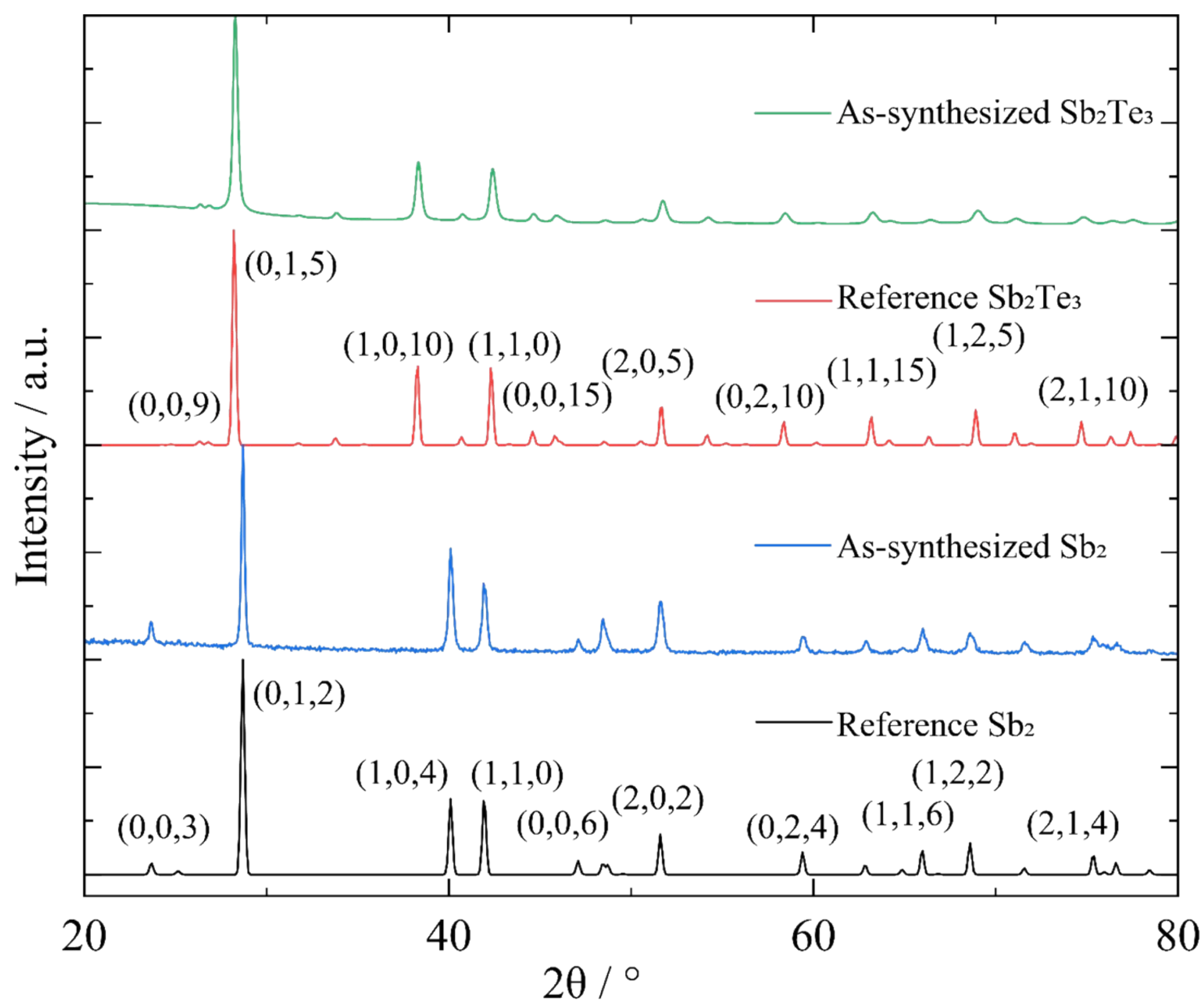

Figure S1. X-ray diffraction of as-synthesized Sb_2Te_3 and Sb_2 with the corresponding reference powder X-ray diffraction data from Inorganic Crystal Structure Database.

Section S3. Correlation Between j_{peak} and the Water Coordination Number for Each Cation

Figure S2 depicts the correlation between coordination numbers for various cations and the peak current density in Figure 3. The coordination numbers for H^+, Na^+, Li^+, and TPA^+ are 3.49, 5.3, 6, and 31,[1] [2-4] respectively.

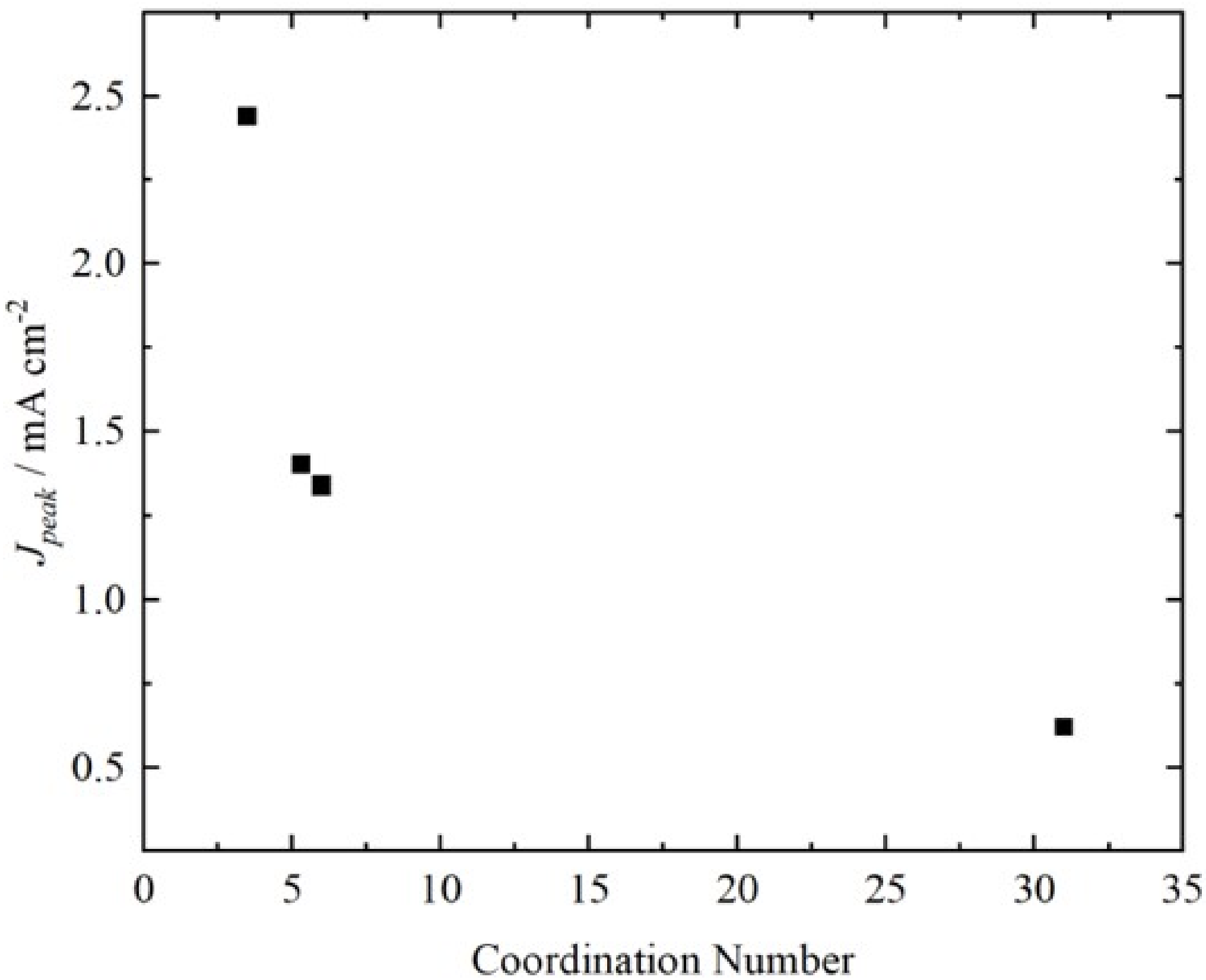

Figure S2. Correlation between the coordination number and j_{peak} using the data shown in the main text and literature values from References S1, S2-S4.

Section S4. Scanning Electron Micrographs of Exfoliated $(Sb_2Te_3)_2(Sb_2)_2$ Flakes Submerged in 0.1 M HCl

Figure S3 presents the SEM images of exfoliated $(Sb_2Te_3)_2(Sb_2)_2$ flakes that were submerged in 0.1 M HCl for 1 hour. After the experiment, the flakes retained a layered morphology with no dissolution of the material in the acidic electrolyte. Althought the contrast between images differed slightly, the terraces visible before and after are unchanged, arguing against appreciable dissolution/etching.

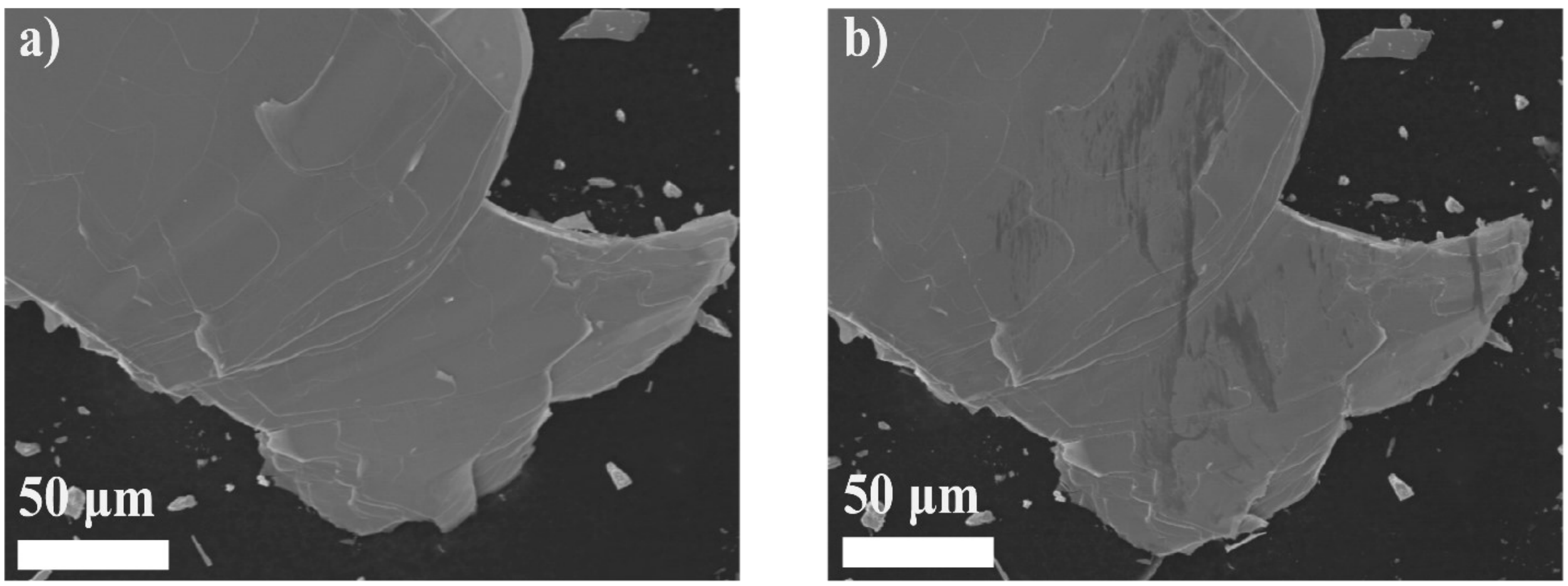

Figure S3. Scanning electron micrographs of exfoliated flakes before (a) and after (b) being submerged in 0.1 M HCl after 1 hour.

Section S5. Atomic Force Microscopy Results of Exfoliated $(Sb_2Te_3)_2(Sb_2)_2$

Figure S4a describes the thinnest structure of exfoliated $(Sb_2Te_3)_2(Sb_2)_2$ as a result of exfoliation at -2.0 V vs. Ag/AgCl in 0.1 M LiCl. The exfoliated flakes were washed and rinsed, then suspended in IPA to form an ink solution. The solution was cast on Si wafer for AFM measurement. The structure's thickness was 4.5 nm, which was equivalent to 6 layers of $(Sb_2Te_3)_2(Sb_2)_2$. Figure S4b describes the distribution of flakes' thickness from various cations. The total number of flakes for each cation was 40, and the bin size was 10 nm. Exfoliation using Na^+ and Li^+ led to thinner flakes than TPA^+ and H_3O^+. Average thickness of exfoliated flakes and percent of thin layers for each cation were reported in Table S1.

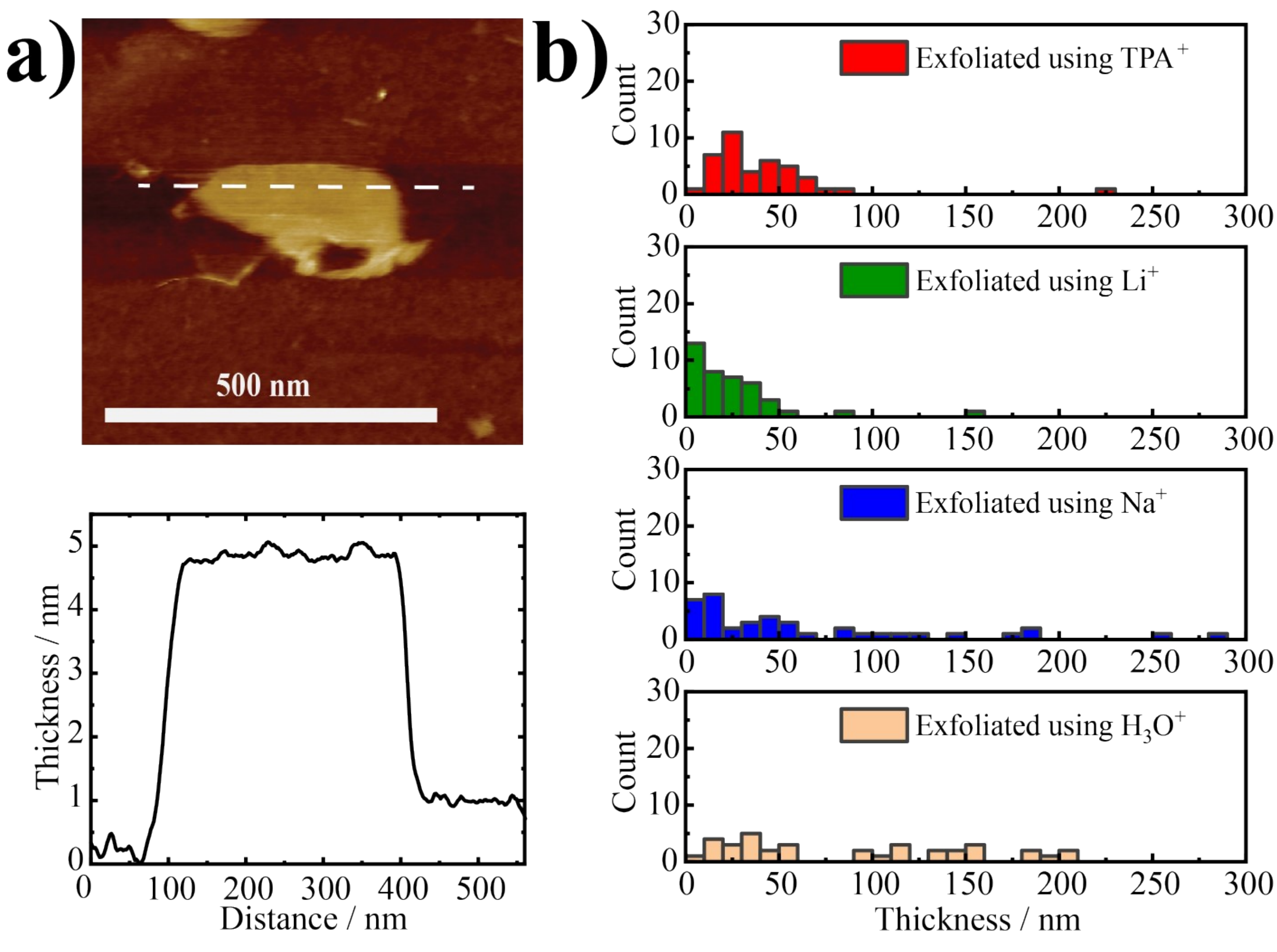

Figure S4. Atomic force microscopy of the thinnest exfoliated flakes using lithium, which exhibited thickness of 4.5 nm (a) and histogram of distribution of flakes' thickness using various cations (b)

Table S1. Average thickness of exfoliated flakes and percent of thin flakes using various cations. Flakes with thickness less than or equal to 10 nm were considered mono- to few layers. Exfoliation using Li^+ yielded the thinnest flakes.

Cations	Average Thickness of Exfoliated Flakes / nm	Percent of Mono-to-Few Layers Flakes / %
TPA^+	41.0 ± 34.8	2.5
Li^+	25.3 ± 26.3	32.5
Na^+	63.6 ± 71.0	17.5
H_3O^+	124.8 ± 131.7	2.5

Section S6. Scanning Electron Micrographs of Exfoliated $(Sb_2Te_3)_2(Sb_2)_2$ Cast on Si Wafer

Figure S5 describes top-down scanning electron micrographs of exfoliated $(Sb_2Te_3)_2(Sb_2)_2$ using various cations cast on a Si wafer. The exfoliated flakes here were the result of exfoliation at -2.0 V vs. *E*(Ag/AgCl) in 0.1 M TPACl, 0.1 M NaCl, 0.1 M LiCl, and 0.1 M HCl. The exfoliated flakes were washed and rinsed, then suspended in IPA to form an ink solution. The results here indicate that due to differences in cation size, the exfoliated flakes exhibited different distributions of lateral dimensions. Specifically, the largest cations yielded a broader distribution of lateral length. In other words, larger cations led to flakes with larger lateral length.

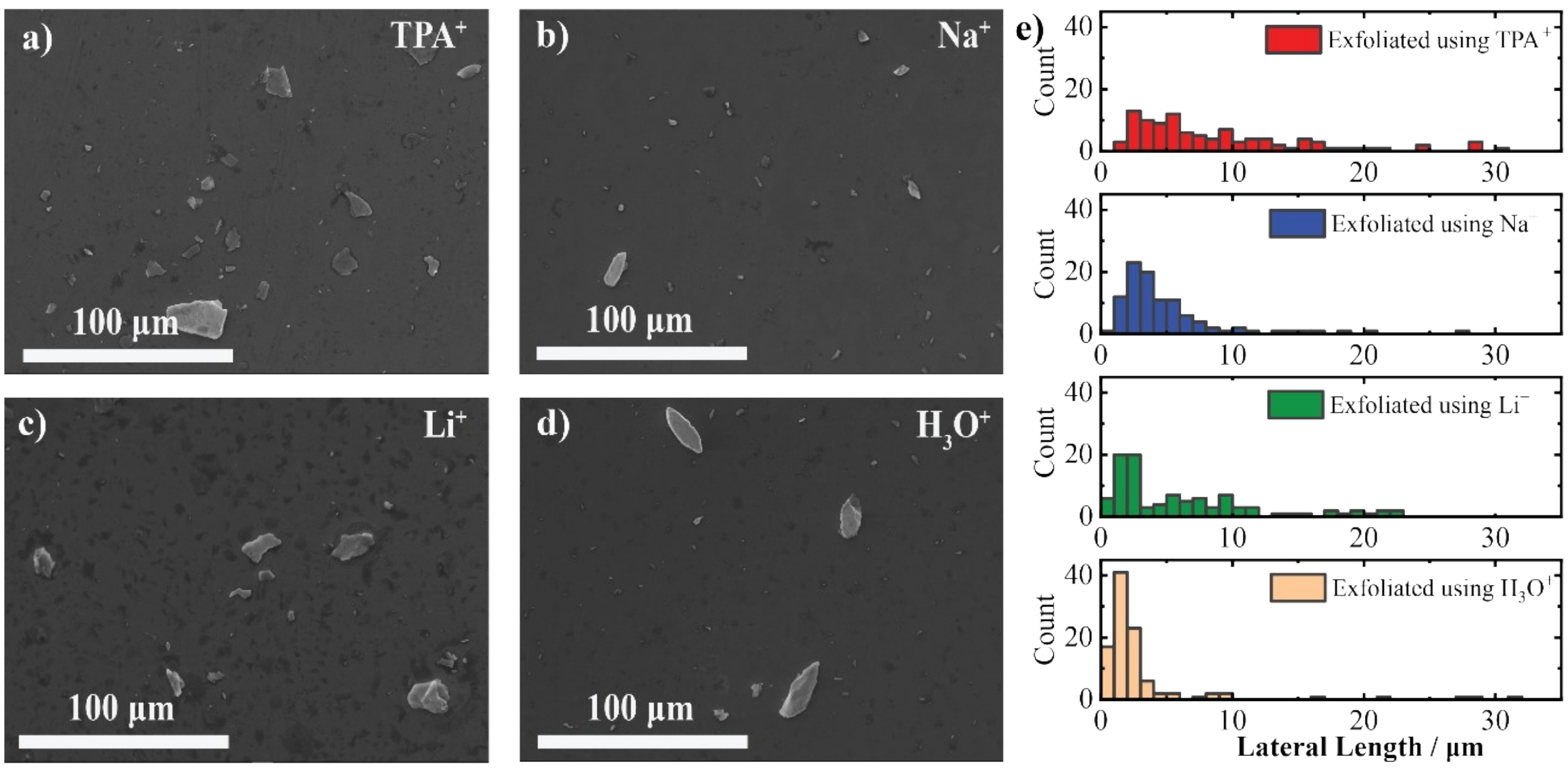

Figure S5. Top-down scanning electron micrographs of exfoliated $(Sb_2Te_3)_2(Sb_2)_2$ using (a) TPA^+, (b) Na^+, (c) Li^+,(d) H^+ cast on Si wafer and (e) histogram denoting the size distribution of these flakes.

Section S7. Electron Diffraction of an Ordered Superlattice Structure

Figure S6 presents the selected area electron diffraction of exfoliated $(Sb_2Te_3)_2(Sb_2)_2$ materials using TPA^+. The structure exhibited kinematically forbidden peaks looking down along the [001] zone axis. When the stage was being tilted slightly away from the [001] zone axis, those kinematically forbidden peaks remained, which confirms that those forbidden peaks were formed not due to dynamic scattering, but, potentially, an ordered superlattice structure.

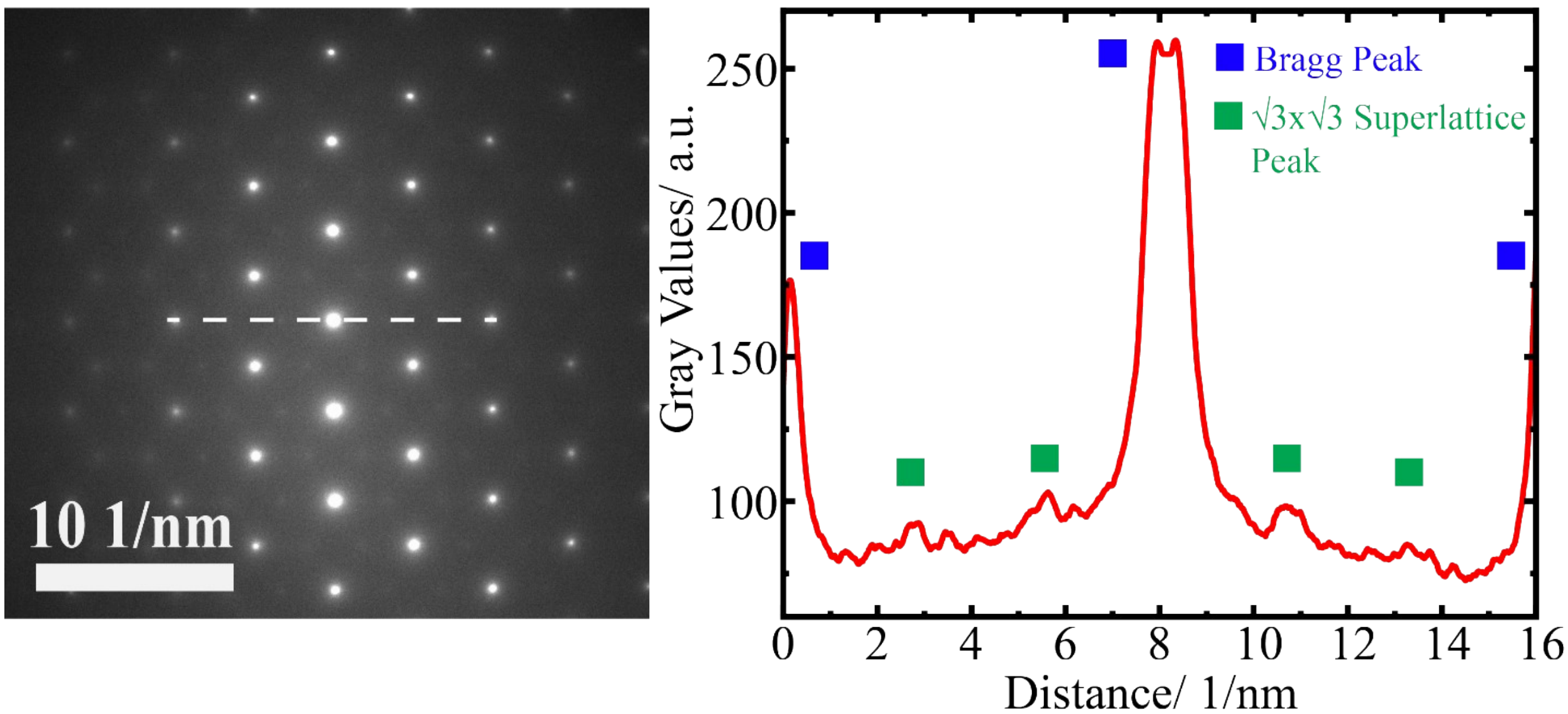

Figure S6. Electron diffraction of $(Sb_2Te_3)_2(Sb_2)_2$ exfoliated using TPA^+ which was obtained at slightly tilted off [001] zone axis and the corresponding gray value profile plot over Braggs and superlattice diffraction spots.

Section S8. Electron Diffraction of Minority Lithiated Products

Figure S7 presents the selected area electron diffraction of byproducts due to lithiation of $(Sb_2Te_3)_2(Sb_2)_2$ in 0.1 M LiCl. The formation of these products was consistent with the electrochemical conversion of Li in Sb_2Te_3 reported in the literature at -2.0 V vs. Ag/AgCl. Only a small number of these byproducts were found, which indicates that the majority of the flakes underwent electrochemical intercalation rather than electrochemical conversion.

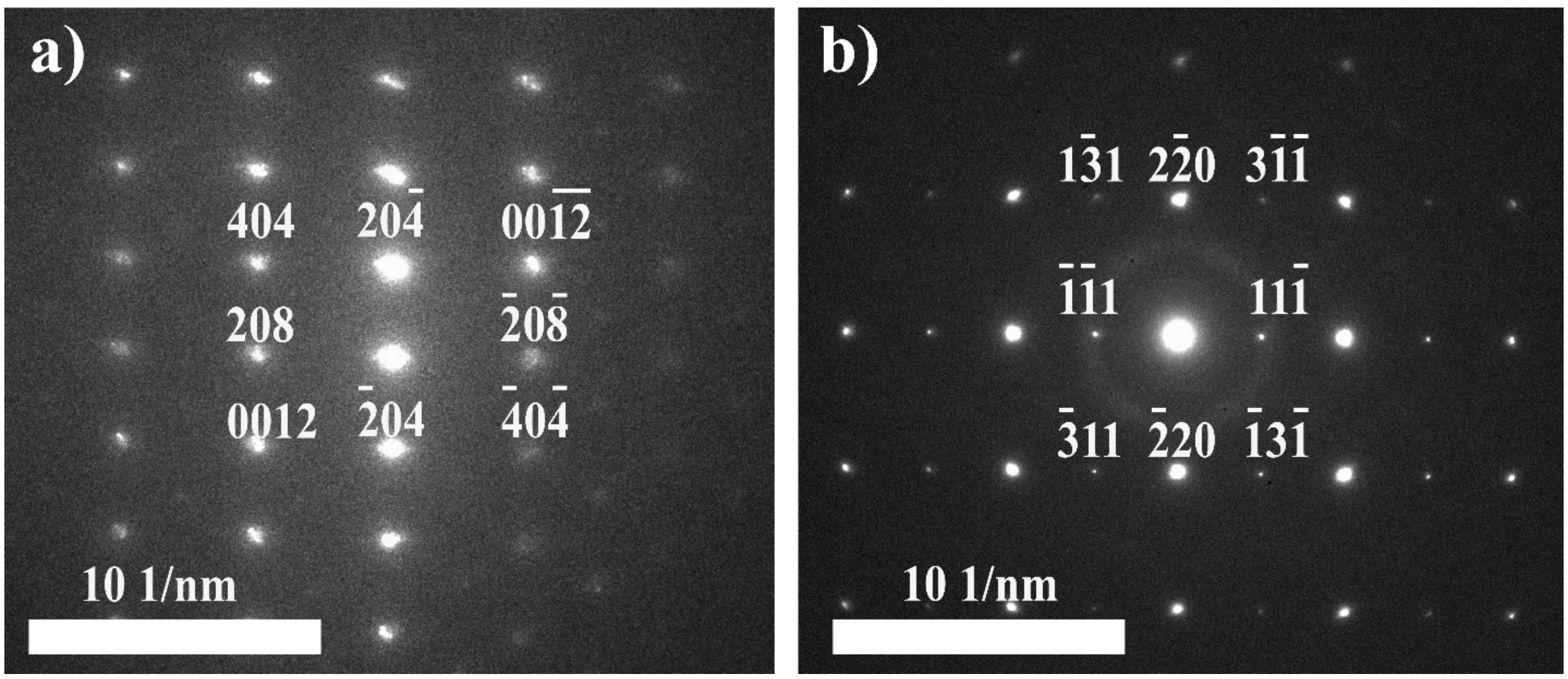

Figure S7. Selected area electron diffraction (SAED) of byproducts of $(Sb_2Te_3)_2(Sb_2)_2$ in 0.1 M LiCl*(aq)* at -2.0 V vs. Ag/AgCl: $LiTe_3$ (a) and $LiSbTe_2$ (b).

Section S9. Raman Spectra of Electrochemically Exfoliated After 1 Year

Figure S8 descibes Raman spectra of different exfoliated flakes measured immediately after and 1 year after preparation. The aged sample was stored in air. The Raman spectra indicated the presence of A1 and E2 modes of Te, the A1g mode of (Sb_2Te_3), and phonons of $SbO_{x.}$ While the relative intensity of A1 Te to other phonons decreased over time, the phonons of SbO_x did not significantly increase in intensity, which indicated the material stable against further oxidation.

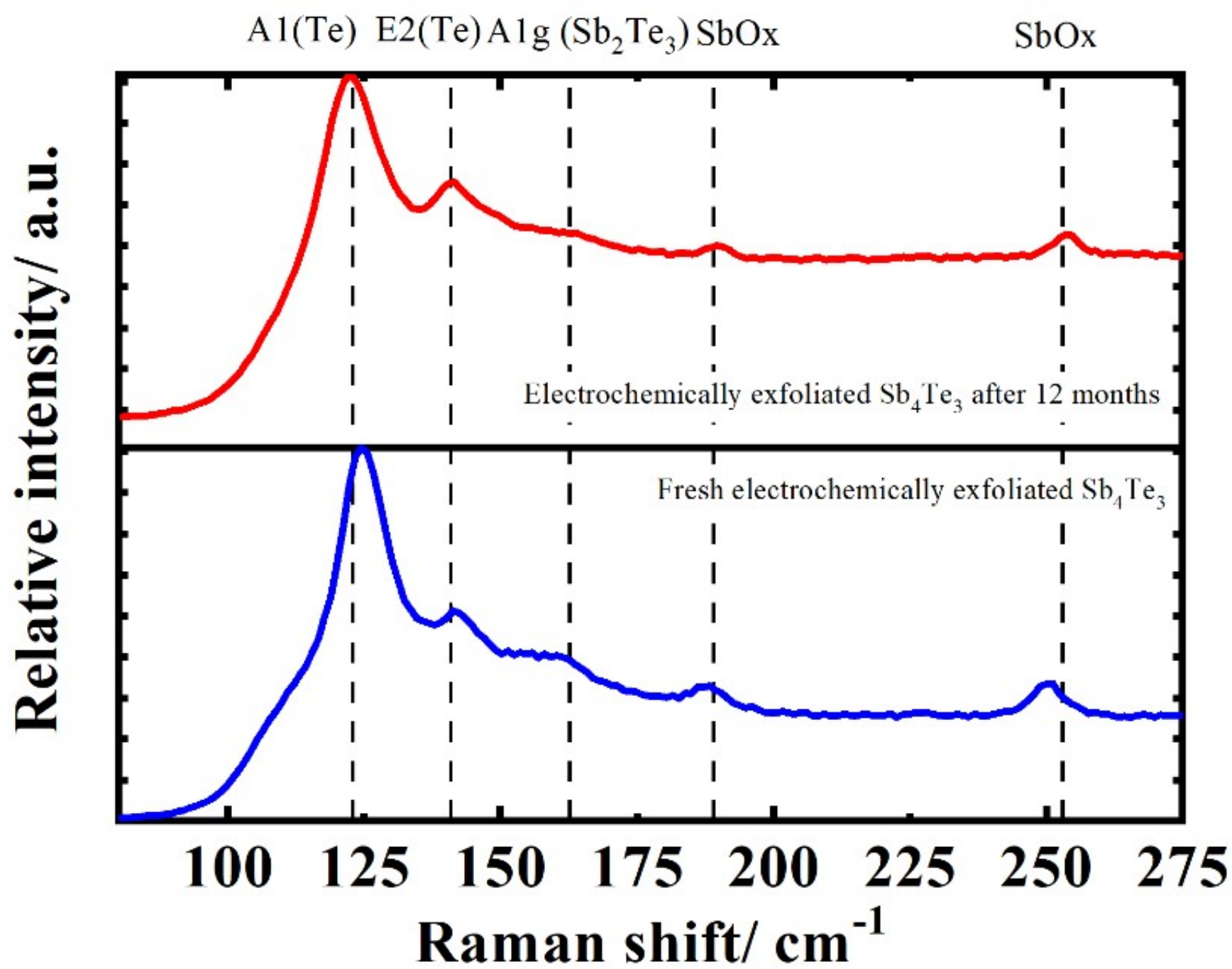

Figure S8. Raman spectra of electrochemical flakes that were exfoliated on June 2025. Raman of exfoliated flake was collected readily after the experiment (blue line). After a year, Raman of different exfoliated flake from the same batch was collected (red line).

Section S10. X-ray Photoelectron Spectra of $(Sb_2Te_3)_2(Sb_2)_2$ Before and After Exfoliation

Figure S9 presents the XPS data of Sb and Te 3d spectra of bulk $(Sb_2Te_3)_2(Sb_2)_2$ (a) and (b) before being applied -2.0 V vs. Ag/AgCl and exfoliated $(Sb_2Te_3)_2(Sb_2)_2$ (c) and (d) after the experiment. scan of the Sb 3d region revealed doublet regions for 3d 5/2 at 528.3 eV and 3d 3/2 at 537.7 eV. Antimony oxide doublet peaks were also observed: 3d 5/2 at 530.2 eV and 3d 3/2 at 539.5 eV. Similarly, for Te, in Te 3d region, there were doublet peaks of Te and TeO_2: 572.3 eV (3d 5/2) and 582.7 eV (3d 3/2) of Te; 576.2 eV (3d 5/2) and 586.6 eV (3d 3/2) of TeO_2. These oxide peaks were greatly reduced in intensity for electrochemically exfoliated flakes. To be specific, the area ratio between the Sb_2O_3 peaks and Sb peaks (0.49) were much lower than those of the bulk $(Sb_2Te_3)_2(Sb_2)_2$ before electrochemical exfoliation (1.77). No tellurium oxide peaks were observed for exfoliated flakes, while Te 3d 3/2 and 3d 5/2 were 583.2 eV and 572.8 eV. The suppression of oxide XPS peaks confirmed that oxides on $(Sb_2Te_3)_2(Sb_2)_2$ were electrochemically reduced at negative applied bias.

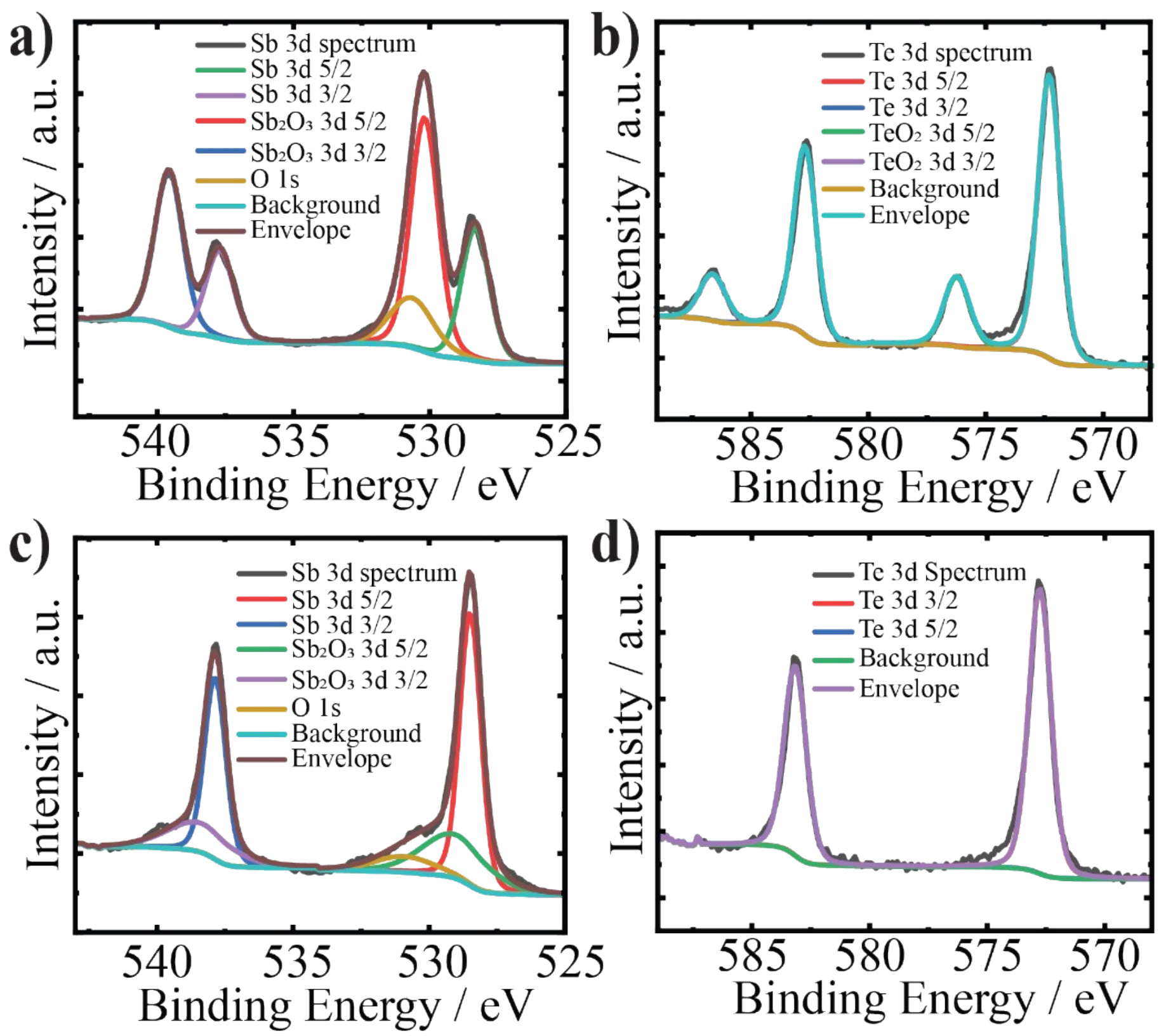

Figure S9. X-ray photoelectron spectra of a,c) Sb 3d and b,d) Te 3d of bulk $(Sb_2Te_3)_2(Sb_2)_2$ a,b) before and c,d) after applying -2.0 V vs. *E*(Ag/AgCl) for exfoliated $(Sb_2Te_3)_2(Sb_2)_2$.

Section S11. Elemental Mapping of Electrochemically Exfoliated Flakes

Figure S10 represents the elemental composition mapping of electrochemically exfoliated $(Sb_2Te_3)_2(Sb_2)_2$ using 0.1 M NaCl. The exfoliated flakes exhibited signals for both Sb and Te. According to Figure S10 a, the Sb:Te composition, obtained from STEM – EDS, for the exfoliated flakes in 0.1 M NaCl was 1.36, which was close to the Sb:Te ratio in the starting materials (1.33). Similarly, the Sb:Te ratio over the large area of the exfoliated flakes casted on Si was 1.33 (Figure S10b).

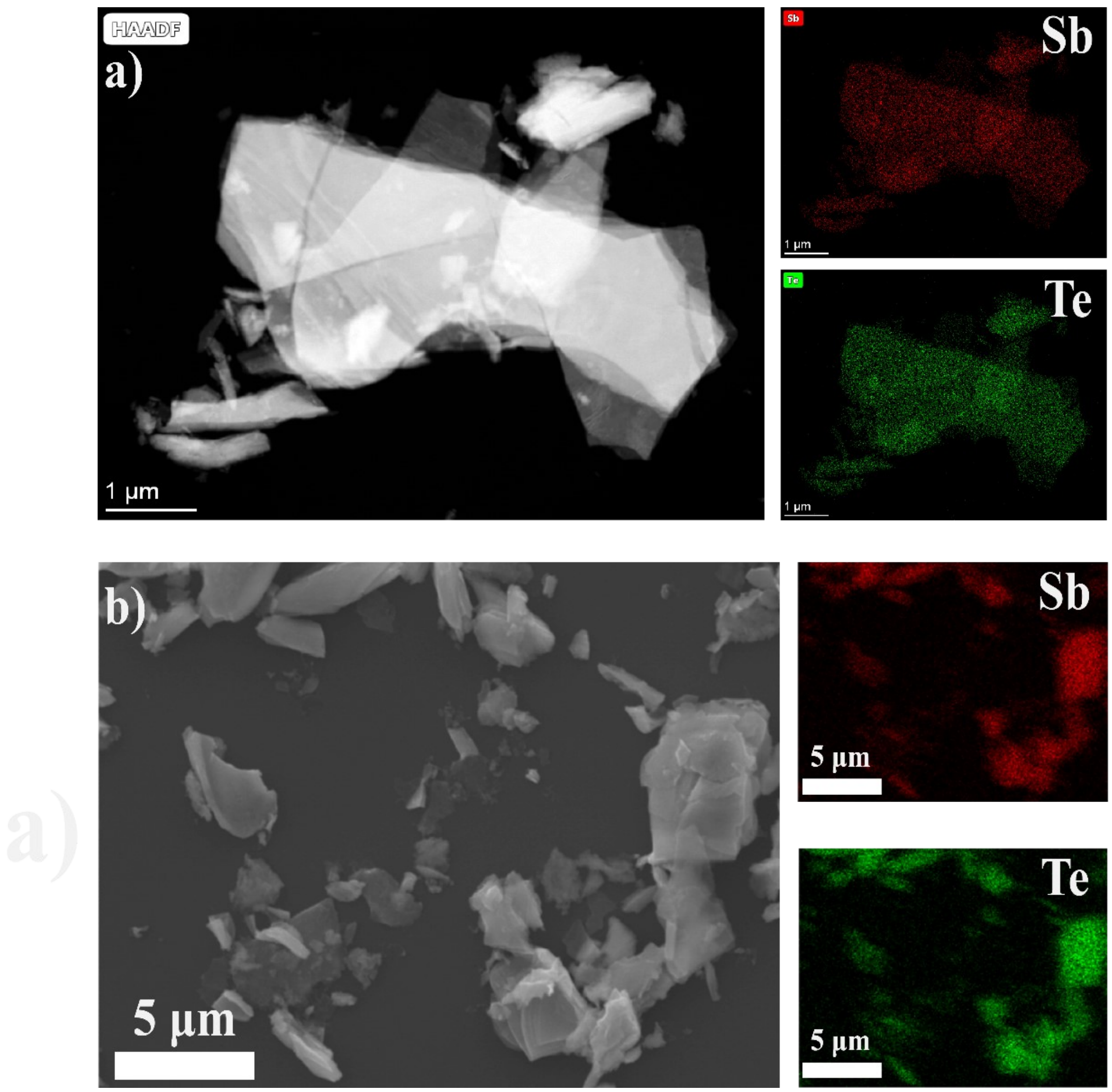

Figure S10. Scanning transmission electron microscopy energy dispersive spectroscopy (STEM – EDS) mapping (a) and scanning electron microscopy energy dispersive spectroscopy (SEM – EDS) mapping (b) of electrochemically exfoliated $(Sb_2Te_3)_2(Sb_2)_2$ flake in 0.1 M NaCl

S12. References

(1) Lounasvuori, M.; Sun, Y. Y. L.; Mathis, T. S.; Puskar, L.; Schade, U.; Jiang, D. E.; Gogotsi, Y.; Petit, T. Vibrational signature of hydrated protons confined in MXene interlayers. *Nat Commun* **2023**, *14* (1). DOI: 10.1038/s41467-023-36842-0.
(2) Bankura, A.; Carnevale, V.; Klein, M. L. Hydration structure of salt solutions from molecular dynamics. *J Chem Phys* **2013**, *138* (1). DOI: 10.1063/1.4772761.
(3) Kameda, Y.; Maeda, S.; Amo, Y.; Usuki, T.; Ikeda, K.; Otomo, T. Neutron Diffraction Study on the Structure of Hydrated Li in Dilute Aqueous Solutions. *J Phys Chem B* **2018**, *122* (5), 1695-1701. DOI: 10.1021/acs.jpcb.7b12218.
(4) Nilsson, E. J.; Alfredsson, V.; Bowron, D. T.; Edler, K. J. A neutron scattering and modelling study of aqueous solutions of tetramethylammonium and tetrapropylammonium bromide. *Phys Chem Chem Phys* **2016**, *18* (16), 11193-11201. DOI: 10.1039/c6cp01389a.
